# Evaluation of Chiral Organosulfur Compounds on Their Activity against the Malaria Parasite *Plasmodium falciparum*

**DOI:** 10.3390/tropicalmed7120416

**Published:** 2022-12-03

**Authors:** Che Julius Ngwa, Rabea Stratmann, Jean Pierre Musabyimana, Kristina Pannen, Jan-Hendrik Schöbel, Marcus Frings, Ingo Schiffers, Calogero Quaranta, Steffen Koschmieder, Nicolas Chatain, Gabriele Pradel, Carsten Bolm

**Affiliations:** 1 Division of Cellular and Applied Infection Biology, Institute of Zoology, RWTH Aachen University, 52074 Aachen, Germany; 2 Department of Hematology, Oncology, Hemostaseology, and Stem Cell Transplantation, Faculty of Medicine, RWTH Aachen University, 52074 Aachen, Germany; 3 Center for Integrated Oncology, Aachen Bonn Cologne Düsseldorf (CIO ABCD), 52074 Aachen, Germany; 4 Institute of Organic Chemistry, RWTH Aachen University, 52074 Aachen, Germany

**Keywords:** malaria, *Plasmodium falciparum*, organosulfur compounds, enantiomers, drugs

## Abstract

Malaria is one of the deadliest tropical diseases, especially causing havoc in children under the age of five in Africa. Although the disease is treatable, the rapid development of drug resistant parasites against frontline drugs requires the search for novel antimalarials. In this study, we tested a series of organosulfur compounds from our internal library for their antiplasmodial effect against *Plasmodium falciparum* asexual and sexual blood stages. Some active compounds were also obtained in enantiomerically pure form and tested individually against asexual blood stages of the parasite to compare their activity. Out of the 23 tested compounds, 7 compounds (**1**, **2**, **5**, **9**, **15**, **16**, and **17**) exhibited high antimalarial activity, with IC_50_ values in the range from 2.2 ± 0.64 to 5.2 ± 1.95 µM, while the other compounds showed moderate to very low activity. The most active compounds also exhibited high activity against the chloroquine-resistant strain, reduced gametocyte development and were not toxic to non-infected red blood cells and Hela cells, as well as the hematopoietic HEL cell line at concentrations below 50 µM. To determine if the enantiomers of the active compounds display different antimalarial activity, enantiomers of two of the active compounds were separated and their antimalarial activity compared. The results show a higher activity of the (–) enantiomers as compared to their (+) counterparts. Our combined data indicate that organosulfur compounds could be exploited as antimalarial drugs and enantiomers of the active compounds may represent a good starting point for the design of novel drugs to target malaria.

## 1. Introduction

Malaria, although a treatable disease in most cases, is a major health problem which threatens nearly half of the world’s population and resulted in over 241 million infections and 627,000 deaths in 2020 [1]. Malaria tropica caused by *Plasmodium falciparum* is considered the deadliest form of the disease, and the eradication of malaria has been hampered by several factors, including the rapid development of drug resistant parasites against front line drugs used in the treatment of infected individuals. There have been several reports of parasite resistance against artemisinin-combination therapies (ACTs) which are the drugs of choice for the treatment of malaria tropica [2,3,4]. This has created an urgent need for the search for and development of novel antimalarials.

Organosulfur compounds are a vital class of molecules with sulfur-containing functional groups, such as sulfones, sulfondiimines, disulfides, sulfoxides, thiophenes, etc. Organosulfur compounds are known to have exceptional properties as free radical scavengers [5]. Natural products such as garlic (*Allium sativum*) are rich in organosulfur compounds and have been shown to display promising antifungal [6], anticancer [7], anti-inflammatory [8]), antibacterial [9,10] and antimalarial activity [11,12]. The major problem with the use of natural products for disease treatment is the dosage, since the composition of the extracts will depend on a number of factors. For example, in the case of garlic, factors such as the source, age, storage conditions and processing method greatly affect the composition of the active ingredients [8]. In addition, the purification of the active compounds from natural sources is very challenging, and most of them are highly volatile and unstable, as has been reported for allicin, an active organosulfur-containing substance from garlic [13,14]. Synthesized derivatives of the compounds may not only improve their stability, but also their activity and solubility.

In this study, in our effort to identify novel antimalarials, we screened various stable organosulfur compounds from our internal compound library and evaluated their effect against the deadliest malaria parasite, *P. falciparum.*

## 2. Materials and Methods

### 2.1. Organosulfur Compounds

**Figure 1:**
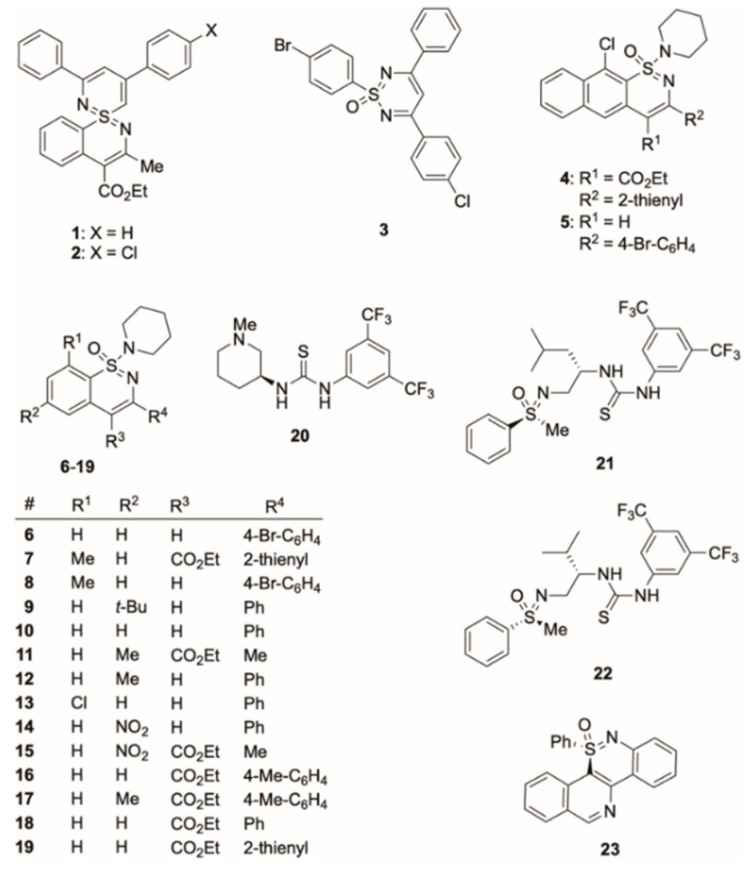
Structures of tested organosulfur compounds.

The tested organosulfur compounds had been prepared in our laboratories and were taken from the internal compound library. Their syntheses and experimental data are already described in the literature [15,16,17,18,19,20,21] Their structures are depicted in Figure 1.

### 2.2. Parasite Culture

Three *P. falciparum* strains were used for compound testing; a strain sensitive to chloroquine (CQ) 3D7, CQ-resistant strain Dd2 and *P. falciparum* gametocyte producing NF54 strain. The 3D7 and Dd2 strains were used to investigate the effect of the compounds on asexual blood stage replication. They were cultured in RPMI 1640/HEPES medium (Gibco, Thermo Scientific, Waltham, MA, USA) containing 10 µg/mL gentamicin (Gibco Thermo Scientific, Waltham, MA, USA,), 50 µg/mL hypoxanthine (Sigma-Aldrich, St. Louis, MO, USA), 0.5% *v*/*v* Albumax II (Gibco, Thermo Scientific, Waltham, MA, USA) and 5% hematocrit. Cultures were maintained at 37 °C using a gas mixture containing 5% CO_2_, 5% O_2_ and 90% N_2_. The NF54 strain was maintained in the same condition and medium as the 3D7 and Dd2 strain, except for the replacement of Albumax II with 10% inactive human serum [22]. To obtain highly synchronized cultures, parasite cultures consisting mainly of ring stages were centrifuged, the pellet was resuspended in 10 mL of 5% *w*/*v* sorbitol/ddH20 and incubated for 10 min at room temperature [23]. After washing the cells with RPMI to remove residual sorbitol, they were again cultivated, as described above. Erythrocyte concentrate and serum samples were obtained from the Department of Transfusion Medicine, University Hospital Aachen, Germany. The serum samples were mixed and pooled and donors were kept anonymous. The work with blood components from humans was approved by the Ethics commission of the RWTH University Hospital (EK 007/13).

### 2.3. Malstat Assay

The antiplasmodial effect of the compounds on *P. falciparum* asexual blood stage development was carried out using the Malstat assay, as described previously [24,25,26]. Compounds dissolved in dimethyl sulfoxide (DMSO) were added to 1% ring stage *P. falciparum* 3D7 and Dd2 cultures (200 µL/well) in a 96-well plate in triplicate at final concentrations ranging from 100 µM to 0.78 µM. The final DMSO concentration in the mixture did not exceed 0.5% *v*/*v*. CQ at concentrations ranging from 500 nM to 4 nM was used as an internal control in the experiments, while parasites treated with 0.5% *v*/*v* DMSO served as the negative control. The parasites were cultured with the compounds for 72 h at 37 °C in the presence of the following gas combination: 5% O_2_, 5% CO_2_, and 90% N_2_. Afterwards, 20 µL of the incubated culture was removed and 100 µL of Malstat reagent (0.1% *v/v* Triton X-100, 1 g of L-lactate, 0.33 g Tris and 33 mg of 3-acetylpyridine adenine dinucleotide in 100 mL of distilled water, pH 9.0) was added in a new 96-well microtiter plate. Next, 20 µL mixture of NBT/Diaphorase (1:1; 1 mg/mL stock each) was added to the Malstat reaction and the parasite lactate dehydrogenase activity was then quantified by measuring the optical densities at 630 nm. IC_50_ values were then determined from variable-slope sigmoidal dose-response graphs using the GraphPad Prism program version 5 (GraphPad Software Inc., La Jolla, San Diego, CA, USA).

### 2.4. Gametocyte Toxicity Test

*P. falciparum* NF54 parasites were cultured at high parasitemia to induce gametocyte formation. Once stage II gametocytes appeared, 1 mL of culture was aliquoted in triplicate in a 24-well plate with compounds at the respective IC_50_ and IC_90_ concentrations that was determined from Malstat assay. The gametocytes were again cultivated for 9 d with the daily replacement of medium. For the first 48 h of cultivation, the gametocytes were treated with test compounds, and subsequently compound free medium was used. On day 9, blood smears were prepared and stained with Giemsa and the gametocytemia was determined by counting the numbers of gametocytes stages IV and V in a total number of 1000 erythrocytes.

### 2.5. Hemolytic Assay

Since the malaria parasite replicates in the red blood cell, we determined if the compounds have an effect on non-infected erythrocytes using hemolytic assays. To this end, 1 mL of washed uninfected erythrocytes at 5% hematocrit in regular medium was plated in triplicate in a 96-well plate (200 µL/well) in the presence of compounds at IC_50_ and IC_90_ concentrations. Erythrocytes treated with 0.15% *w/v* saponin were used as lysis control and 0.5% *v/v* DMSO was used as the negative control. Following 48 h of incubation at 37 °C, the plate was centrifuged at 500× *g* for 2 min and 100 µL of the supernatant was transferred to another plate and the absorbance was measured at 550 nm with a spectrophotometer.

### 2.6. Cytotoxicity Assay

The MTT [3-(4,5-dimethylthiazol-2-yl)-2,5-diphenyltetrazolium bromide] assay was performed using the human cervical cancer cell (Hela) line and the hematologic malignancy human erythroleukemia (HEL) cell line to determine if the compounds were toxic (loss of viability) against human cells. MTT is taken up by viable cells and converted by mitochondrial dehydrogenases to a violet formazan product that cannot diffuse through cell membranes, and therefore crystallizes in viable cells [27]. The adherent Hela cells were plated in 96-well microtiter plates and cultured at 37 °C overnight. Compounds were added to the cultures in different serial concentrations in triplicate the next day, and the cells were incubated for 72 h. DMSO was used as the negative control at a final concentration of 0.5% *v/v*, and a concentration of 15% *v/v* DMSO which is toxic was added as the positive control. After 72 h of drug treatment, medium-containing drug was replaced with 100 µL fresh drug-free medium. Then, 20 µL MTT (5 mg/mL) was added to each well and incubated for 3 h at 37 °C. The medium was subsequently removed and 100 µL solubilization solution (5% *w/v* SDS, 0.1 M HCl in 100% vol. DMSO) was added and incubated for 30 min under rotation at room temperature to solubilize the formazan crystals.

As described before [27], the suspension-cultured HEL cells were plated in 96-well microtiter plates at a density of 2 × 10^5^ cells/100 µL RPMI 1640 + 10% FCS + 5% P/S per well. The compounds dissolved in DMSO were added at concentrations ranging from 1 to 200 µM. Controls were treated with DMSO (0.5%). The measurement of cell viability was performed 72 h later, using 10 µL MTT reagent, subsequent incubation in the dark at 37 °C for 4 h, and for solubilization, 100 µL isopropanol-HCl solution per well was added.

For both cell lines, the optical density (OD) of the solution was measured at 550 nm. The OD-values of cultures treated with compounds were normalized to the OD-values of 0.5% *v/v* DMSO control (set to 100% viability for Hela or 1 for HEL cell line). IC_50_ values were calculated from MTT assays using Graphpad Prism (V9).

## 3. Results

### 3.1. Organosulfur Compounds

The compounds have either been described in the scientific literature or their preparation and analysis is detailed in the Supplementary Materials (Figures S1–S19). In general, all compounds have been fully characterized and their structures can be found in Figure 1.

### 3.2. The Organic Compounds Exhibit Antimalarial Activity

We first investigated the 23 compounds for their antiplasmodial activities against the CQ-sensitive 3D7 strain of *P. falciparum* using the Malstat assay. The IC_50_ of each compound was then determined and used to estimate the 90% inhibitory concentration (IC_90_). As the positive control, CQ was used in the assays. We observed that 7 out of the 23 compounds, **1**, **2**, **5**, **9**, **15**, **16**, and **17**, displayed antiplasmodial activities with IC_50_ values at lower micromolar ranges (2.2 ± 0.64 µM to 5.2 ± 1.95 µM; Table 1). Six other compounds, **4**, **6**, **8**, **11**, **14**, **20**, and **21**, showed moderate antiplasmodial activity with IC_50_ values ranging from 8 ± 0.53 µM to 14.1 ± 1.50 µM. The remaining compounds showed either very low antiplasmodial activity or they were inactive (Table 1).

The four most active compounds (**5**, **9**, **16**, and **17**) were then tested against the CQ-resistant Dd2 strain. All four compounds were highly active against the strain, although the antiplasmodial activity was slightly higher than for the 3D7 strain (Table 1). As expected, a significant decrease in antiplasmodial activity was observed for CQ against Dd2 compared to 3D7.

### 3.3. The (–) Enantiomers Exhibit Better Antiplasmodial Activity as Compared to Their (+) Counterparts

**Figure 2:**
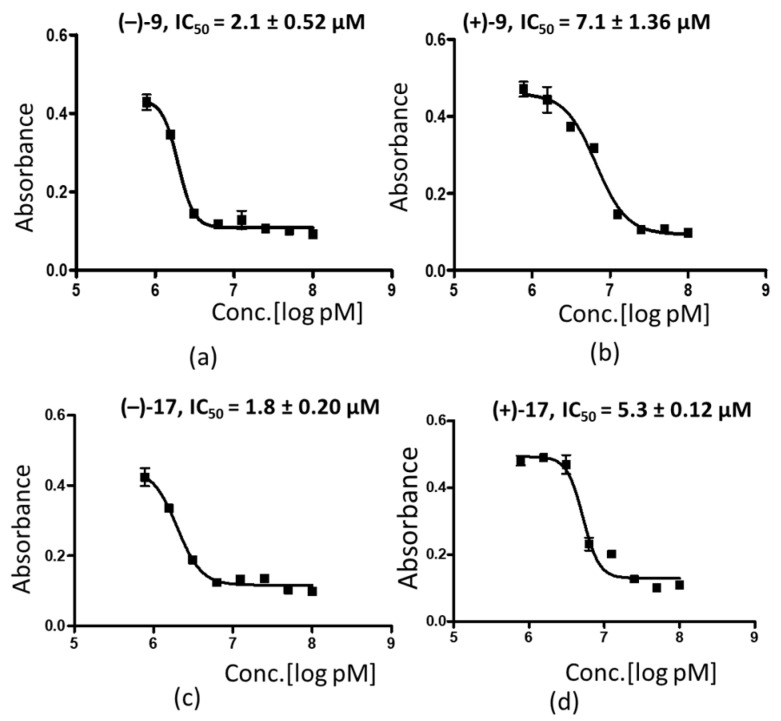
Antiplasmodial activity of the enantiomers of compounds **9** and **17** on *P. falciparum.* Logarithmic dose response curves for the testing of the enantiomers (**a**) (–)-**9**, (**b**) (+)-**9**, (**c**) (–)-**17** and (**d**) (+)-**17** on the *P. falciparum* 3D7 strain. Ring stage parasites were cultured with the compounds at concentrations of 100 µM to 0.78 µM for 72 h at 37 °C. CQ served as control. The viability of the parasites was determined by measuring the plasmodial LDH activity using the Malstat assay, and the absorbance of the photometric reaction was measured at OD_630_ nm. The figures are representative of one of three independent experiments.

To determine if single stereoisomers of the compounds could improve the antiplasmodial activity, we resynthesized compounds **9** and **17** and separated their enantiomers. Then, the antiplasmodial activity of each enantiomer was evaluated using the Malstat assay. The results show an improved activity for (–)-**9** and (–)-**17** compared to their mirror images (+)-**9** and (+)-**17** (Figure 2a–d). It is noteworthy that the isolated (–) compounds were slightly more active when compared to their racemic mixtures (2.1 ± 0.52 µM vs. 3.3 ± 1.56 µM for compound **9** and 1.8 ± 0.20 vs. 2.6 ± 0.70 µM for **17**; Table 1, Figure 2).

### 3.4. Treatment with Compounds Reduce Gametocyte Development

**Figure 3:**
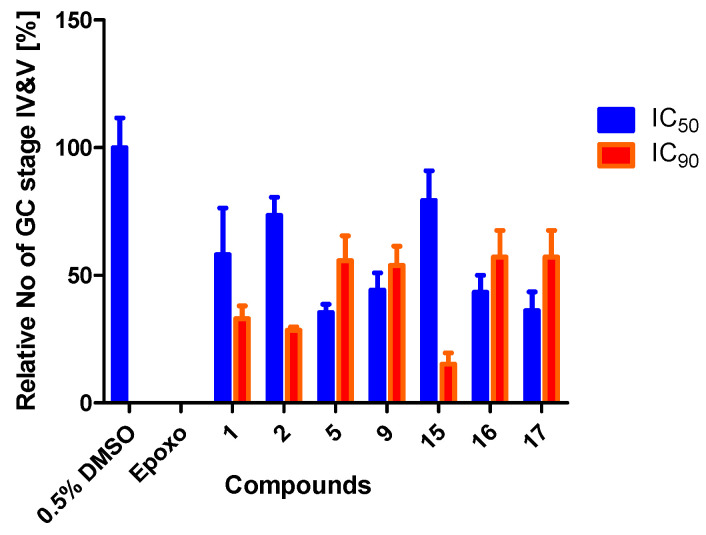
Effect of organosulfur compounds on gametocyte development. Cultures rich in stage II gametocyte were incubated with compounds at IC_50_ and IC_90_ concentrations for 2 d, and again cultivated for another 7 d with daily replacement of medium. The numbers of stage IV and V gametocytes were determined from Giemsa smears and normalized to 0.5% *v*/*v* DMSO treatment which served as negative control (set to 100%). Epoxomicin (Epoxo) at 60 nM served as positive control.

An optimal drug should be able to target not only the asexual blood stages of the parasite, which causes the clinical manifestation of the disease, but also the gametocytes, which are responsible for the transmission of the disease to the mosquito vector. To determine if the organosulfur compounds have an effect on gametocyte development, we incubated a stage II gametocyte-rich culture with compounds **1**, **2**, **5, 9**, **15**, **16**, and **17** at IC_50_ and IC_90_ concentrations for 48 h and the gametocytes were fed with drug-free medium for another 7 d, before Giemsa smears were prepared and the gametocyte development was followed. As the positive control, the proteasome inhibitor epoxomicin (60 nM) was used, while DMSO (0.5% *v*/*v*) served as the negative control in the experiments. Following the addition of the compounds at their IC_50_ and IC_90_ concentrations, compounds **1**, **2**, and **15** showed a very high reduction in stage IV and V gametocyte numbers at IC_90_ concentration, while the other showed a slight reduction. At IC_50_ concentrations, compounds **5**, **9**, **16**, and **17** reduced mature gametocyte numbers by about 50% while compounds **1**, **2**, and **15** showed only minor reduction (Figure 3).

### 3.5. The Compounds Exhibit No Hemolytic or Less Cytotoxic Effect on Human Cells

**Figure 4:**
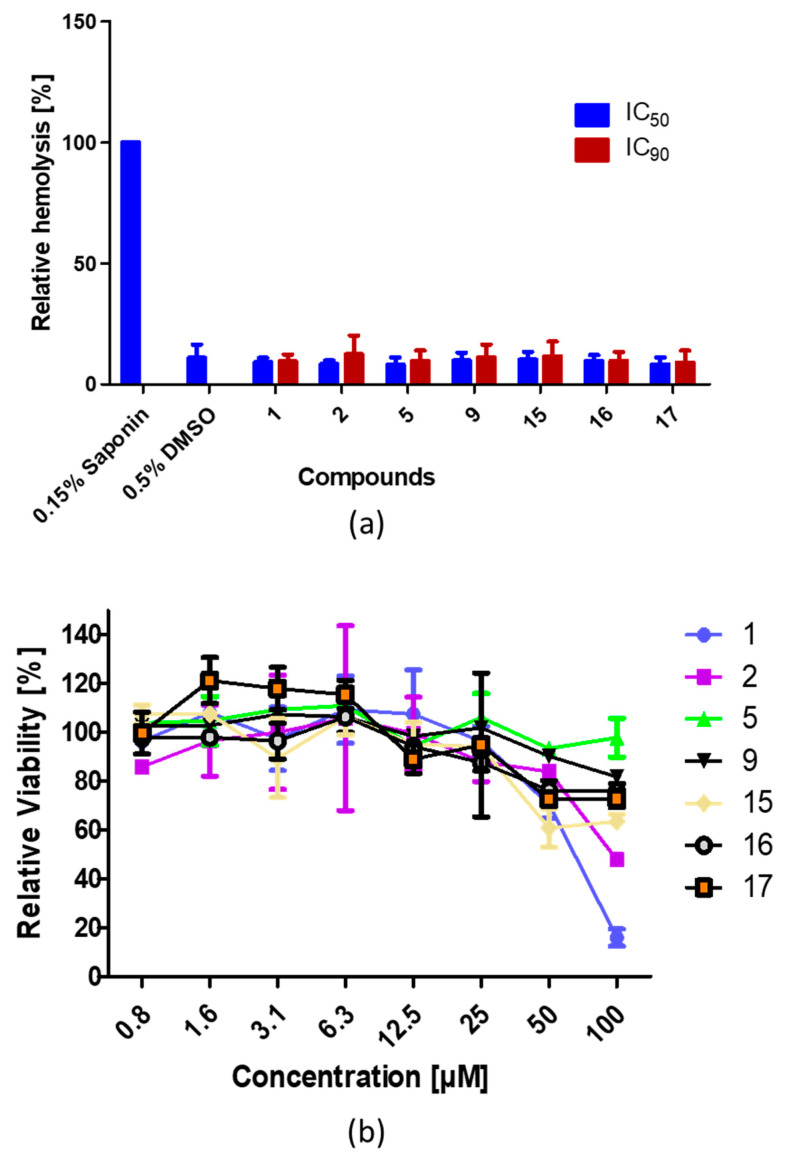
Hemolytic and cytotoxic effect of compounds. (**a**) Hemolytic effect of compounds. Compounds were added to fresh erythrocytes (5% hematocrit) at IC_50_ and IC_90_ concentrations and incubated at 37 °C for 48 h. The absorbance of free hemoglobin was measured at OD_550_ nm in the supernatant and normalized to 0.15% *w*/*v* saponin (set to 100%), which served as positive control in the experiments. 0.5% *v/v* DMSO was used as negative control. (**b**) Cytotoxicity effect of compounds on Hela cells. The cells were plated in 96-well microtiter plates and cultured overnight at 37 °C. The following day, the old medium was removed and 100 µL of fresh medium containing serially diluted compounds in corresponding wells at final concentrations ranging from 100 µM to 0.8 µM was added and the culture was incubated for 72 h. After incubation, the medium was replaced by 100 µL fresh drug-free medium and 20 µL MTT (5 mg/mL) was added in each well and incubated for 3 h at 37 °C. After 3 h, the MTT preparation was then removed from each well and 100 µL solubilization solution was added. Plates were then placed on a rotator at about 500 rpm at RT until the formazan precipitate was completely resuspended and the OD was measured at 450 nm. The data are representative of three independent experiments.

To assess if the compounds have unexpected side effects by lysing non-infected erythrocytes, a hemolysis test was performed. Fresh erythrocytes were incubated with **1**, **2**, **5**, **9**, **15**, **16**, and **17** at IC_50_ and IC_90_ concentrations for 48 h at 37 °C and hemolysis was determined by measuring the amount of hemoglobin released in the cultures spectrophotometrically. Erythrocytes treated with 0.15% *w*/*v* saponin were used as the positive control, while erythrocytes incubated with 0.5% *v*/*v* DMSO or medium served as the negative control. The assay demonstrated no hemolytic effect of all compounds at IC_50_ and IC_90_ concentrations with no differences in extracellular hemoglobin content measurable between compound-treated and 0.5% *v*/*v* DMSO-treated erythrocytes (Figure 4a).

The compounds were further investigated for their cytotoxicity on Hela cells using the MTT assay which measures metabolic activity. Cells were cultured for 72 h with compound **1**, **2**, **5**, **9**, **15**, **16**, and **17** at different concentrations, and cell viability was determined using the MTT assay and compared with that of 0.05% *v*/*v* DMSO which served as the control (set to 100% viability). The results show that at a concentration of about 25 µM more than 90% of the cells were viable, while at 50 µM more than 60% cell viability was obtained (Figure 4b).

### 3.6. Analyzed Enantiomers Were Not Toxic against HEL Cells

**Figure 5:**
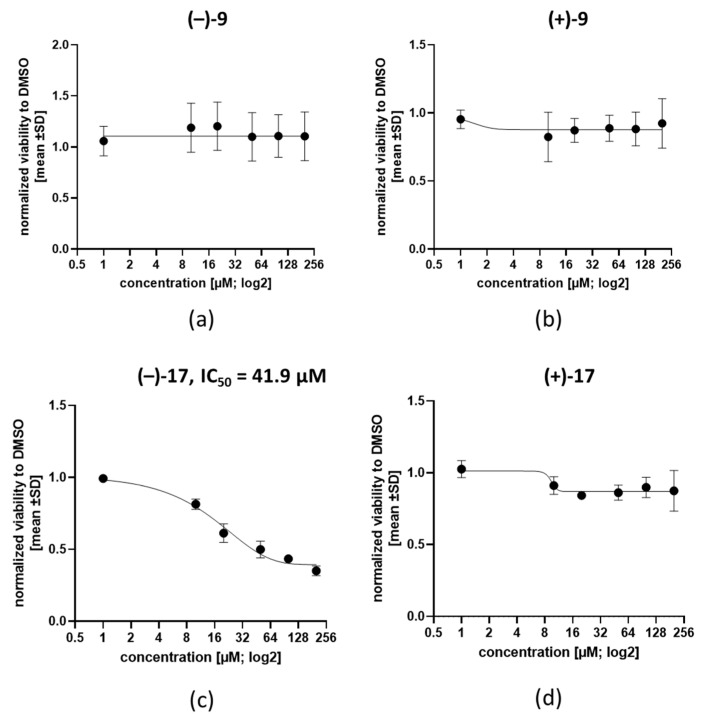
Low cytotoxicity of enantiomers on hematopoietic HEL cells. HEL cells were treated with (–)-**9** (**a**), (+)-**9** (**b**), (–)-**17** (**c**), and (+)-**17** (**d**) at concentrations of 1, 10, 20, 50, 100 and 200 µM for 72 h or DMSO as control. All data were normalized to the DMSO control and shown as mean ± SD. Graphs show a combination of three independent experiments.

When the cytotoxicity of the enantiomers was determined against HEL cells, only (–)-**17** showed an EC_50_ of 41.9 µM (Figure 5c) with a selectivity index of 23.2, while the other enantiomers were mainly not toxic at the tested concentrations (Figure 5a,b,d).

## 4. Discussion

Malaria continues to be one of the most devastating infectious tropical diseases in the world, especially due to the rapid development of drug resistant parasites, limited efficacy of the lone vaccine, the RTS, S [28,29] and high risk of aggravation of disease transmission due to climate change [30]. New drugs are therefore needed to overcome these obstacles. Organosulfur compounds have a history in traditional medicine and natural products rich in organosulfur compounds, such as garlic and onion, have high medicinal value [5,31]. In this study, we tested a range of organosulfur molecules for their antimalaria activity against *P. falciparum.* We show that some of the organosulfur compounds act effectively against the asexual blood stages of both the CQ-sensitive and -resistant strain of *P. falciparum* at low micromolar concentrations. These results confirm previous findings, which demonstrated the antimalarial effect of both natural products rich in organosulfur compounds, such as garlic [11,12], and organosulfur molecules synthesized in the laboratory [32,33]. Some of the tested compounds also reduced gametocyte development, thereby indicating that they may be exploited as multistage drugs which will not only elevate the clinical manifestation of malaria, but also reduce the transmission of the disease from the human to the mosquito.

The investigated sulfur compounds can be categorized into two classes: sulfur-based heterocycles and thioureas. Two compounds of the latter class (**21** and **22**) contain sulfoximidoyl units as well. Interestingly, relevant antimalarial activities were only observed for compounds with sulfur-based heterocycles. Among them were the two novel spirocyclic sulfondiimines **1** and **2**. Furthermore, 1-(piperidin-1-yl) naphtho-1,2-thiazine 1-oxide **5** and structurally related 1-amino-1,2-benzothiazine 1-oxides **9**, **15**, **16**, and **17** proved active. Comparing those molecules to inactive compounds (see Figure 1 and Table 1) revealed structural differences, but no apparent molecular group or entity could be identified to be responsible for the detected antimalarial activities. For example, in the aforementioned series of 1-amino-1,2-benzothiazine 1-oxides (**9**, **15**, **16**, and **17**), the benzo group could be unsubstituted or bear either electron-donating or -withdrawing substituents at position 6 (here R^2^). Closely related structures, however, with very similar substitution pattern (as, for example, **10** or **11**) were inactive. Besides position 6 of the 1-amino-1,2-benzothiazine 1-oxides other positions (such as 3, here R^4^) showed the same variability. In both active and inactive molecules, R^4^ could be an alkyl or an aryl group. To predict which was best for achieving antimalarial activity has still remained impossible. In the currently investigated compound library, all 1,2-benzothiazine 1-oxides had a 1-(piperidin-1-yl) substituent, which was kept constant in order to allow for determining the influence of other molecular impacts. In future work, this amino group shall be varied leading to a broader variability of the investigated substrate space.

We also demonstrated that the enantiomers of the two active compounds **9** and **17** showed a different antimalarial activity, indicating that the spatial arrangement of the atoms in the molecular skeleton played a vital role in their antimalarial activity. This observation is in line with previous work, where similar effects have been observed [34,35]. As the single enantiomers of **9** and **17** showed a higher activity than their racemic mixtures, future studies of the antimalarial effects of related chiral compounds must involve a preceding resolution leading to single enantiomers which can then be tested individually. The exact mode of action of the herein reported organosulfur compounds shall be elucidated in future studies.

Finally, and importantly, the compounds studied here showed no significant toxicity against non-infected red blood cells and other human cells indicating that they are safe and mainly target the parasite-infected red blood cells.

## 5. Conclusions

This study reports on the antimalarial- evaluation of organosulfur compounds available from our compound library. We show the high antimalaria activity of seven compounds (**1, 2, 5, 9, 15, 16,** and **17**) with IC_50_ values ranging from 2.2 ± 0.64 to 5.2 ± 1.95 µM. Two compounds, **9** and **17**, were also tested as single enantiomers against asexual blood stages of the parasite to compare their activity which showed a better activity for the (–) when compared to the (+) enantiomer. Our combined data therefore confirm that these organosulfur compounds are active against the malaria parasite and that their single enantiomers represent promising starting points for the design of novel antimalarial drugs.

## Figures and Tables

**Table 1:** Antiplasmodial effect of compounds against the CQ-sensitive 3D7 and CQ-resistant Dd2 strains of *P. falciparum*.

Compound	IC_50_ (µM) CQ-Sensitive Strain 3D7	IC_90_ (µM)	IC_50_ (µM) CQ-Resistant Strain Dd2
**1**	**3.8 ± 0.66**	34.2	
**2**	**3.12 ± 0.58**	27.9	
**3**	22.1±1.06		
**4**	9.1 ± 2.59		
**5**	**2.2 ± 0.64**	19.8	**6.8 ± 1.54**
**6**	10.6 ± 1.53		
**7**	35.8 ± 5.11		
**8**	12.5 ± 3.27		
**9**	3.3 ± 1.56	29.7	**6.5 ± 0.87**
**10**	46.4 ± 2.67		
**11**	12 ± 4.28		
**12**	21.3 ± 4.05		
**13**	20.3 ± 1.69		
**14**	10.3 ± 0.90		
**15**	**5.2 ± 1.95**	46.8	
**16**	**3.1 ± 0.26**	27.9	**4.4 ± 0.59**
**17**	**2.6 ± 0.70**	23.4	**4 ± 0.28**
**18**	26.5 ± 1.19		
**19**	31.5 ±4.9		
**20**	8 ± 0.53		
**21**	14.1 ± 1.50		
**22**	15.9 ± 1.46		
**23**	24.95 ± 1.06		
**Chloroquine**	0.02 ± 0.004		0.14 ± 0.063

Note: IC_50_ values of most active compounds are indicated in bold.

## Data Availability

Not applicable.

## Supplementary Materials

The following supporting information can be downloaded at: https://www.mdpi.com/article/10.3390/tropicalmed7120416/s1, SI (Figures S1–S19): Chemical syntheses, analytical data including NMR spectra and HPLC chromatograms of new compounds. References [36,37,38] are cited in the Supplementary Materials.
