# Supplementary material for: Evaluation of Chiral Organosulfur Compounds on Their Activity against the Malaria Parasite Plasmodium falciparum
Source: Trop Med Infect Dis. 2022 Dec 3;7(12):416. doi: 10.3390/tropicalmed7120416 (PMC9785921; doi:10.3390/tropicalmed7120416)
Supplement: Supplementary file 1 [file tropicalmed-07-00416-s001.zip › tropicalmed-2012138-supplementary.pdf]

Supporting Information

Evaluation of chiral organosulfur compounds on their activity against the malaria parasite *Plasmodium falciparum*

Che Julius Ngwa[1]*, Rabea Stratmann[1], Jean Pierre Musabyimana[1], Kristina Pannen[2,3], Jan-Hendrik Schöbel[4,5], Marcus Frings[4], Ingo Schiffers[4], Calogero Quaranta[4], Steffen Koschmieder[2,3], Nicolas Chatain[2,3], Gabriele Pradel[1], and Carsten Bolm[4]*

[1]Division of Cellular and Applied Infection Biology, Institute of Zoology, RWTH Aachen University, Worringerweg 1, 52074 Aachen, Germany

[2] Department of Hematology, Oncology, Hemostaseology, and Stem Cell Transplantation, Faculty of Medicine, RWTH Aachen University, Aachen, Germany

[3] Center for Integrated Oncology, Aachen Bonn Cologne Düsseldorf (CIO ABCD), Aachen, Germany

[4] Institute of Organic Chemistry, RWTH Aachen University, Aachen, Germany

[5] New address: NUVISAN ICB GmbH, Muellerstr. 178, 13353 Berlin, Germany

* Correspondence: ngwa.che@bio2.rwth-aachen.de, Carsten.Bolm@rwth-aachen.de

Table of Contents

1. General information…… S1
2. Analytical data…… S2
3. References…… S7
4. NMR spectra…… S9
5. CSP-HPLC chromatograms…… S14

1. General information

Reaction mixtures were stirred with a magnetic stirring bar and heated with a silicon-oil bath using a magnetic stirring and heating plate. For reactions under inert atmosphere, standard Schlenk techniques were used, with argon as inert gas.

All reagents were purchased from commercial sources and used without further purification. The solvents *n*-hexane, ethyl acetate and dichloromethane (DCM) were purified by distillation prior using. The solvents for NMR analysis were also purchased and used without further purification. Anhydrous solvents were purchased and stored over molecular sieves. The solvents *i*-propanol and ethanol were purchased in HPLC grade purity from Sigma-Aldrich or Fisher-Scientific. All other solvents were used in technical grade purity.

Known substances **1**–**22** were taken from our internal compound library. Their syntheses and analytical data have already been described elsewhere.[1-5] As far as we can judge with the NMR spectra, the purities of the applied compounds have been ≥95%. Newly available enantiomers or novel compounds have been characterized in full.

Mechanochemical reactions were performed in a Retsch MM400 mixer mill using stainless steel (SS) milling jars with a volume of 5 mL equipped with two SS balls with the diameter of 7 mm.

Thin layer chromatography (TLC) was performed using TLC plates with silica gel 60M coated aluminum plates from Merck with fluorescence indicator F_{254}. For the detection of the substances on the TLC plates UV light (λ = 254 nm) was used. For flash column chromatography silica gel 60 (40–63 µm) from Macherey-Nagel and purified sea sand (0.3 mm) from Grüssing was used. Nitrogen gas was used to apply pressure (0.1–0.4 bar) during flash column chromatography. The mixing ratios of the eluents were measured by volume.

All analytical high performance liquid chromatography (HPLC) separations were performed on an Agilent 1200 HPLC system with a chiral stationary phase (AD-H-column, length: 250 mm, diameter: 4.6 mm) from Chiralpak. The enantiomeric excess (*ee*) of a compound was calculated by the ratio of enantiomers in the HPLC chromatogram. Racemic mixtures of compounds were separated by preparative HPLC on a chiral stationary phase (CSP). A Varian SD-1 solvent-pump with a ProStar 320 UV-detector was used for all preparative HPLC. For the separation of enantiomers from a racemic mixture was used either a (AD-column, length: 250 mm, diameter: 50 mm) or a (OD-column, length: 250 mm, diameter: 50 mm) from Universalpack.

The measurements of optical rotations were conducted on a Krüss P3000 polarimeter with a monochromatic sodium lamp (λ = 589 nm). The specific rotation $[\alpha]_D^{RT}$ is given in the unit deg·mL·g^{-1}·dm^{-1} and the concentration *c* is given in the unit g·$(100mL)^{-1}$.

NMR spectra were recorded at room temperature on Varian VNMRS 400, Varian VNMRS 600 or Bruker Avance Neo 600 spectrometers and processed and analyzed with the program MestReNova. The chemical shifts δ are given in parts per million (ppm) relative to the residual non-deuterated solvent peaks for ^{1}H NMR spectra [$CHCl_3$: δ_H = 7.26 ppm, CH_2Cl_2: δ_H = 5.32 ppm] and relative to the deuterated solvent peaks for $^{13}C\{^1H\}$ NMR spectra [$CDCl_3$: δ_C = 77.16 ppm, CH_2Cl_2: δ_C = 53.84 ppm]. The chemical shifts in ^{13}C NMR were rounded to the first decimal place, unless a greater precision was needed to distinguish closely spaced peaks. The multiplicity of the signals was reported with the following abbreviation: s (singlet), d (doublet), t (triplet), q (quartet), m (multiplet), br (broad signal) and combinations of these. The coupling constants *J* were reported in the unit Hertz (Hz).

Mass spectra [electron ionization (EI), 70 eV; chemical ionization (CI), methane, 100 eV] were recorded on a Finnigan SSQ 7000 spectrometer and high-resolution mass spectrometry (HRMS) were recorded on a Thermo Fisher Scientific LTQ Orbitrap XL spectrometer [electrospray ionization (ESI) in positive ion mode].

Infrared (IR) spectra were recorded using the attenuated total reflectance (ATR) technique on a Perkin Elmer 100 FT-IR spectrometer with a UATR Diamond KRS-5 unit. Wave numbers *v* are given in the unit cm^{-1}.

The melting point (mp) was determined on a Büchi B-540 melting point apparatus using open-end capillary tubes.

2. Analytical Data

Ethyl 3-methyl-3',5'-diphenyl-1λ^6-spiro[benzo[*e*][1,2]thiazine-1,1'-[1,2]thiazine]-4-carboxylate (1)

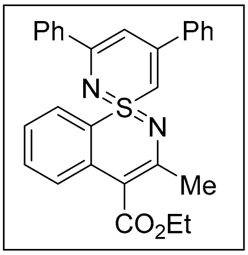

Title compound **1** has already been described in the literature.[1] The NMR data are presented here, since these data are not accessible elsewhere.

^{1}H NMR (600 MHz, $CDCl_3$): δ = 7.92–7.86 (m, 2H), 7.74–7.67 (m, 3H), 7.59 (dd, *J* = 8.1, 1.4 Hz, 1H), 7.56–7.52 (m, 1H), 7.51–7.46 (m, 3H), 7.40–7.35 (m, 4H), 6.57 (s, 1H), 5.70 (s, 1H), 4.41 (q, *J* = 7.2 Hz, 2H), 2.37 (s, 3H), 1.42 (t, *J* = 7.2 Hz, 3H) ppm. **$^{13}C\{^1H\}$ NMR (151 MHz, $CDCl_3$):** δ = 169.1, 150.5, 150.3, 149.2, 138.9, 138.4, 133.8, 133.1,

130.0, 129.8, 129.1 (2C), 128.9, 128.5 (2C), 127.5 (4C), 126.5, 123.3, 116.6, 104.1, 96.1, 82.7, 61.0, 25.1, 14.5 ppm.

Ethyl 5'-(4-chlorophenyl)-3-methyl-3'-phenyl-1λ^6-spiro[benzo[*e*][1,2]thiazine-1,1'-[1,2]thiazine]-4-carboxylate (2)

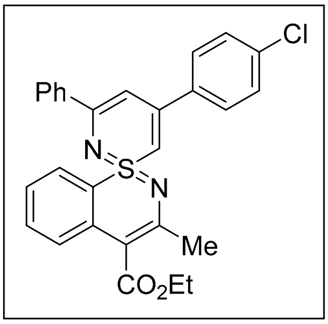

Title compound **2** has already been described in the literature.[1] The NMR data are presented here, since these data are not accessible elsewhere.

^{1}H NMR (600 MHz, CDCl$_3$): δ = 7.90–7.85 (m, 2H), 7.70 (d, *J* = 7.4 Hz, 1H), 7.66–7.60 (m, 2H), 7.59–7.56 (m, 1H), 7.56–7.53 (m, 1H), 7.47–7.44 (m, 2H), 7.41–7.34 (m, 4H), 6.53–6.48 (m, 1H), 5.65 (s, 1H), 4.41 (q, *J* = 7.2 Hz, 2H), 2.36 (s, 3H), 1.42 (t, *J* = 7.2 Hz, 3H) ppm. **^{13}C{^{1}H} NMR (151 MHz, CDCl$_3$):** δ = 169.1, 150.5, 149.1, 149.0, 138.2, 137.3, 136.2, 133.9, 133.2, 129.9, 129.3 (2C), 128.9, 128.8 (2C), 128.6 (2C), 127.5 (2C), 126.6, 123.3, 116.4, 104.2, 95.7, 82.5, 61.0, 25.1, 14.5 ppm.

6-(*tert*-Butyl)-3-phenyl-1-(piperidin-1-yl)benzo[*e*][1,2]thiazine 1-oxide (*rac*-9)

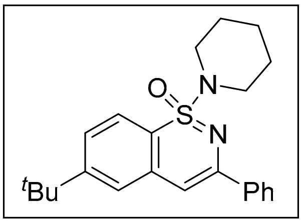

Title compound *rac*-**9** was synthesized according to a published procedure[2]. Because a larger quantity of *rac*-**9** was required for the preparative CSP-HPLC separation, the reaction was repeated three times, and the product was purified in one batch.

A Schlenk tube equipped with a magnetic stirring bar and a septum was loaded with 1-(4-(*tert*-butyl)phenylsulfonimidoyl)piperidine (100 mg, 0.357 mmol, 1.0 equiv.), 2-oxo-2-phenylethyl methanesulfonate (107 mg, 0.499 mmol, 1.4 equiv.), sodium acetate (41.0 mg, 0.499 mmol, 1.4 equiv.), copper(II) acetate (6.5 mg, 36 µmol, 0.1 equiv.) and [Cp*Rh(MeCN)$_3$][SbF$_6$] (14.8 mg, 17.8 µmol, 0.05 equiv.). Afterwards, anhydrous methanol (5 mL, 0.07 M) was added. The resulting mixture was stirred for 13 h at 40 °C under argon atmosphere. The reaction mixture was filtered through a pad of Celite and rinsed with DCM:MeOH (10:1). The solvents were removed under reduced pressure and the products of the three reactions were purified (in one batch) by flash column chromatography on silica gel (*n*-pentane:EtOAc, 5:1) to obtain *rac*-**9** (279 mg, 0.732 mmol, 69%, based on three reactions) as a yellow oil.

^{1}H NMR (400 MHz, CDCl$_3$): δ = 8.00–7.94 (m, 2H), 7.66 (d, J = 8.5 Hz, 1H), 7.45–7.31 (m, 5H), 6.59 (s, 1H), 3.02–2.97 (m, 4H), 1.69–1.55 (m, 4H), 1.55–1.46 (m, 2H), 1.38 (s, 9H) ppm. **^{13}C {^{1}H} NMR (101 MHz, CDCl$_3$):** δ = 155.7, 148.5, 139.4, 138.8, 128.7, 128.4, 126.4, 124.4, 124.0, 122.8, 114.5, 98.0, 46.8, 35.3, 31.2, 25.6, 24.0 ppm. **IR (ATR):** ν = 3187, 3058, 2953, 2852, 2321, 2080, 1936, 1883, 1795, 1592, 1533, 1471, 1451, 1402, 1353, 1289, 1251, 1211, 1151, 1124, 1101, 1046, 956, 914, 881, 835, 752, 707, 685, 655 cm^{-1}. **MS (ESI):** calculated for $C_{23}H_{29}N_2OS^+$: *m/z* = 381.1995 [M + H]$^+$, found: *m/z* = 381.1988.
The analytical data matches the ones previously reported in the literature.[S2]
The enantiomers of the racemic product *rac*-**9** were obtained by a **preparative CSP-HPLC:** [OD-column, *n*-hexane:*i*-PrOH = 98:2, flow = 40 mL·min^{-1}, rt, λ = 254 nm, retention time: 16.6 min (+), 43.1 min (–)]. After the separation, the enantiomers (+)-**9** und (–)-**9** were further purified by three flash column chromatographies on silica gel [1. column (*n*-hexane:DCM, 50:1 → $CHCl_3$:MeOH, 200:1), 2. column (*n*-pentane:EtOAc, 20:1), 3. column (*n*-pentane:DCM, 50:1 → $CHCl_3$:MeOH 200:1]. The enantiomeric excess of the enantiomers was determined by analytical CSP-HPLC:

(+)-**9**
CSP-HPLC: AD-H-column, *n*-heptane:*i*-PrOH = 50:50, flow = 0.5 mL·min^{-1}, 20 °C, λ = 210 nm, retention time: 15.9 min, *ee* > 99%.
$[\alpha]_D^{RT}$ = +66.3 (*c* = 0.8, in $CHCl_3$)
(–)-**9**
CSP-HPLC: AD-H-column, *n*-heptane:*i*-PrOH = 50:50, flow = 0.5 mL·min^{-1}, 20 °C, λ = 210 nm, retention time: 19.2 min (–), *ee* > 99%.
$[\alpha]_D^{RT}$ = –39.6 (*c* = 1.0, in $CHCl_3$)
The absolute values of the specific rotation of (+)-**9** and **(**–)-**9** differ probably due to the non-linearity of the optical rotation with respect to the concentration.

Ethyl 6-methyl-1-(piperidin-1-yl)-3-(*p*-tolyl)-1λ^4-benzo[e][1,2]thiazine-4-carboxylate 1-oxide (*rac*-17)

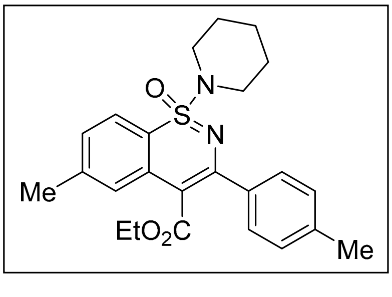

Title compound *rac*-**17** was synthesized according to a published procedure[2]. Because a larger quantity of *rac*-**17** was required for the preparative CSP-HPLC separation, the reaction was performed four times, and the product was purified in one batch.

1-(4-Methylphenylsulfonimidoyl)piperidine (70.0 mg, 0.294 mmol, 1.0 equiv.), ethyl 2-diazo-3-oxo-3-(*p*-tolyl)propanoate (102 mg, 0.441 mmol, 1.5 equiv.), pivalic acid (60.0 mg, 0.587 mmol, 2.0 equiv.),

$[Cp^*IrCl_2]_2$ (5.9 mg, 7.3 µmol, 0.025 equiv.) and silica (230 mg) were loaded into a 5 mm SS-milling jar equipped with two 7 mm milling balls. An argon atmosphere was created inside the milling jar with a cannula attached to a light argon stream before tightly closing the milling jar. The reaction mixture was ground at 25 Hz for 1 h. The product of four batches was purified by flash column chromatography on silica gel (*n*-pentane:EtOAc, 10:1 → 6:1) to obtain *rac*-**17** (320 mg, 0.754 mmol, 64%) as a yellow oil.

^{1}H NMR (600 MHz, $CDCl_3$): δ = 7.67 (d, *J* = 8.2 Hz, 1H), 7.64 (s, 1H), 7.47–7.43 (m, 2H), 7.23 (dd, *J* = 8.1, 1.6 Hz, 1H), 7.19–7.15 (m, 2H), 4.01 (dq, *J* = 10.7, 7.1 Hz, 1H), 3.96 (dq, *J* = 10.7, 7.1 Hz, 1H), 3.07 (ddd, *J* = 11.4, 7.3, 3.7 Hz, 2H), 2.98 (ddd, *J* = 11.4, 7.3, 3.7 Hz, 2H), 2.45 (s, 3H), 2.37 (s, 3H), 1.67–1.53 (m, 4H), 1.53–1.48 (m, 2H), 0.86 (t, *J* = 7.2 Hz, 3H) ppm. **^{13}C {^{1}H} NMR (151 MHz, $CDCl_3$):** δ = 169.8, 153.9, 143.6, 138.7, 138.0, 136.8, 128.8, 128.1, 127.7, 124.2, 124.0, 113.3, 104.8, 61.0, 46.7, 25.5, 23.9, 22.3, 21.4, 13.6 ppm. **IR (ATR):** ν = 2934, 2855, 2158, 2087, 1987, 1910, 1699, 1602, 1563, 1526, 1501, 1464, 1399, 1354, 1319, 1279, 1227, 1185, 1131, 1074, 1038, 936, 814, 756, 707 cm^{-1}. **MS (ESI):** calculated for $C_{24}H_{29}N_2O_3S^+$: *m/z* = 425.1893 [M + H]$^+$, found: *m/z* = 425.1887.

The analytical data matches the ones previously reported in the literature[2].

The enantiomers of the racemic product *rac*-**17** were separated by a **preparative CSP-HPLC:** [AD-column, *n*-hexane:EtOH = 96:4, flow = 40 mL·min^{-1}, rt, λ = 254 nm, retention time: 39.2 min (+), 45.2 min (–)]. Afterwards the enantiomers (+)-**17** and (–)-**17** were purified twice by flash column chromatography on silica [1. column (*n*-pentan:EtOAc, 1:0 → 0:1), 2. column (*n*-pentan:DCM, 50:1 → $CHCl_3$:MeOH 200:1]. The enantiomeric excess was determined by analytical CSP-HPLC:

(+)-**17**

CSP-HPLC: AD-H-column, *n*-heptane:EtOH = 50:50, flow = 0.5 mL·min^{-1}, 20 °C, λ = 210 nm, retention time: 11.1 min (+), 13.1 min (–), *ee* > 99%.

$[\alpha]_D^{RT}$ = +66.5 (*c* = 0.8, in $CHCl_3$)

(–)-**17**

CSP-HPLC: AD-H-column, *n*-heptane:EtOH = 50:50, flow = 0.5 mL·min^{-1}, 20 °C, λ = 210 nm, retention time: 12.2 min (+), 14.1 min (–), *ee* = 98%.

$[\alpha]_D^{RT}$ = –73.3 (*c* = 0.4, in $CHCl_3$)

The absolute values of the specific rotation of **(+)-17** and **(–)-17** differ probably due to the non-linearity of the optical rotation with respect to the concentration.

(*R*)-5-Phenylbenzo[3,4][1,2]thiazino[5,6-*c*]isoquinoline 5-oxide (23)

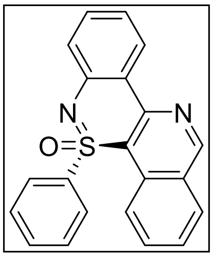

The title compound **23** was obtained by applying a protocol for the reduction of nitriles.[6]

In a Schlenk flask lithium aluminum hydride (95 wt%, 5.0 mg, 0.13 mmol, 1.1 equiv.) was added to anhydrous diethyl ether (5 mL) at 0 °C under an atmosphere of argon. To this stirred suspension (*R*)-(–)-2-[(5-oxido-5-phenyl-5λ^4-isoquino[4,3-*c*][2,1]benzothiazin-12-yl)amino]benzonitrile[7] (52.1 mg, 0.114 mmol) was carefully added in small portions. The reaction mixture continued to stir for 30 min and was then carefully treated with water (1 mL). Subsequently, an aqueous solution (1 mL) of sodium hydroxide (20 wt%) was added. After further dilution with more water (10 mL), the crude product was extracted with dichloromethane from the aqueous phase three times. The combined organic solvents were dried with magnesium sulfate and then removed under reduced pressure. The residue was subjected to flash column chromatography on silica gel (*n*-pentane:EtOAc, 2:1) and the title compound was obtained as yellow solid (17.6 mg, 0.0514 mmol, 45%). Furthermore, unreacted starting material, i.e. (*R*)-(–)-2-[(5-oxido-5-phenyl-5λ^4-isoquino[4,3-*c*][2,1]benzothiazin-12-yl)amino]benzonitrile, was separately recovered by the same flash column chromatography (17.4 mg, 0.0379 mmol, 33%).

mp: 203–205 °C. **^{1}H NMR (600 MHz, CD_2Cl_2):** δ = 9.54 (s, 1H), 8.87 (d, *J* = 8.2 Hz, 1H), 8.16 (d, *J* = 8.2 Hz, 1H), 8.07 (d, *J* = 7.6 Hz, 1H), 7.88 (d, *J* = 8.3 Hz, 2H), 7.67–7.57 (m, 3H), 7.55–7.47 (m, 3H), 7.25 (d, *J* = 7.9 Hz, 1H), 7.20 (t, *J* = 7.5 Hz, 1H) ppm. **^{13}C {^{1}H} NMR (151 MHz, CD_2Cl_2):** δ = 158.26, 148.08, 144.24, 144.06, 133.64, 132.96, 132.44, 130.85, 129.53, 129.51, 128.33, 128.13, 126.59, 123.85, 123.77, 120.98, 118.27, 111.68. **IR (ATR):** ν = 3059, 2923, 2322, 2083, 1678, 1599, 1572, 1542, 1459, 1420, 1378, 1340, 1301, 1245, 1205, 1150, 1127, 1092, 1033, 1006, 978, 851, 801, 751, 684 cm^{-1}. **MS (EI):** *m/z* = 342 (100, $M^{+\bullet}$), 294 (20), 265 (15), 271 (32), 190 (3). **MS (CI):** *m/z* = 343 (100, $[M + H]^+$). **HRMS (ESI):** calculated for $C_{21}H_{14}N_2NaOS^+$: *m/z* = 365.07191 $[M + Na]^+$, found: *m/z* = 365.07187 with Δ = –0.10 ppm.

The optical rotation of compound **23** was not determined. Compound **23** contains a trace amount of grease as impurity (indicated in the ^{1}H NMR spectrum).[8]

Please notice that the originally attempted synthesis of (*R*)-12-{[2-(aminomethyl)phenyl]amino}-5-phenylbenzo[3,4][1,2]thiazino[5,6-*c*]isoquinoline 5-oxide by reducing the nitrile group of the starting material did not succeed, and title compound **23** was isolated as the sole reaction product instead (Figure 1):

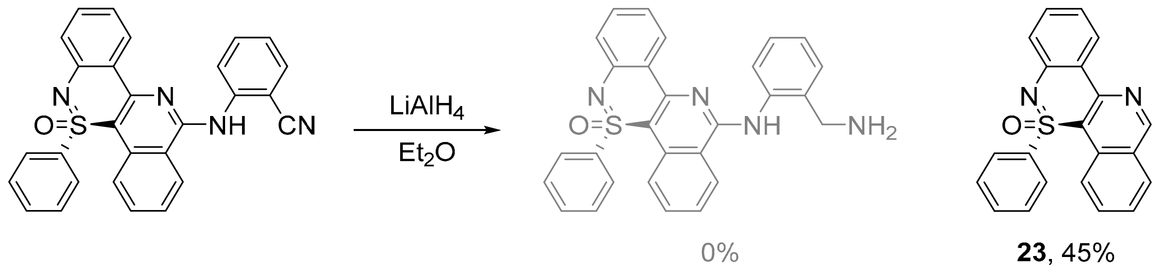

Figure S1: Failed reduction of (*R*)-(–)-2-[(5-oxido-5-phenyl-5λ^4-isoquino[4,3-*c*][2,1]benzothiazin-12-yl)amino]-benzonitrile to (*R*)-12-{[2-(aminomethyl)phenyl]amino}-5-phenylbenzo[3,4][1,2]thiazino[5,6-*c*]isoquinoline 5-oxide gives rise to (*R*)-5-phenylbenzo[3,4][1,2]thiazino[5,6-*c*]isoquinoline 5-oxide (**23**).

3. References

[1] Schöbel, J.-H. Dissertation, RWTH Aachen University (Germany), **2021**, doi:10.18154/RWTH-2021-08148.

[2] Schöbel, J.-H.; Elbers, P.; Truong, K.-N.; Rissanen, K.; Bolm, C. 1,2-Benzothiazine Derivatives from Sulfonimidamides by Metal-Catalyzed Annulation Reactions in Solution and under Solvent-Free Mechanochemical Conditions. *Adv. Synth. Catal.* **2021**, *363*, 1322–1329, doi:10.1002/adsc.202001505.

[3] Schöbel, J.-H.; Passia, M. T.; Wolter, N. A.; Puttreddy, R.; Rissanen, K.; Bolm, C. 1,2,6-Thiadiazine 1-Oxides: Unsaturated Three-Dimensional S,N-Heterocycles from Sulfonimidamides. *Org. Lett.* **2020,** *22*, 2702–2706, doi: 10.1021/acs.orglett.0c00666.

[4] Jörres, M.; Schiffers, I.; Atodiresei, I.; Bolm, C. Asymmetric Michael Additions of α-Nitrocyclohexanone to Aryl Nitroalkenes Catalyzed by Natural Amino Acid-Derived Bifunctional Thioureas. *Org. Lett.* **2012**, *14*, 4518–4521, doi:10.1021/ol302005f.

[5] Frings, M.; Thomé, I.; Bolm, C. Synthesis of Chiral Sulfoximine-Based Thioureas and Their Application in Asymmetric Organocatalysis. *Beilstein J. Org. Chem.* **2012**, *8*, 1443–1451, doi:10.3762/bjoc.8.164.

[6] Amundsen, L. H.; Nelson, L. S. Reduction of Nitriles to Primary Amines with Lithium Aluminum Hydride. *J. Am. Chem. Soc.* **1951**, *73*, 242–244, doi:10.1021/ja01145a082.

[7] Frings, M.; Atodiresei, I.; Bolm, C. (*R*)-(–)-2-[(5-Oxido-5-phenyl-5λ^4-isoquino[4,3-*c*][2,1]-benzothiazin-12-yl)amino]benzonitrile. *Molbank* **2014**, M834; doi:10.3390/m834.

[8] Fulmer, G. R.; Miller A. J. M.; Sherden, N. H.; Gottlieb, H. E.; Nudelman, A.; Stoltz, B. M.; Bercaw, J. E.; Goldberg, K. I. NMR Chemical Shifts of Trace Impurities: Common Laboratory

Solvents, Organics, and Gases in Deuterated Solvents Relevant to the Organometallic Chemist. *Organometallics* **2010**, *29*, 2176–2179, doi:10.1021/om100106e.

4. NMR Spectra

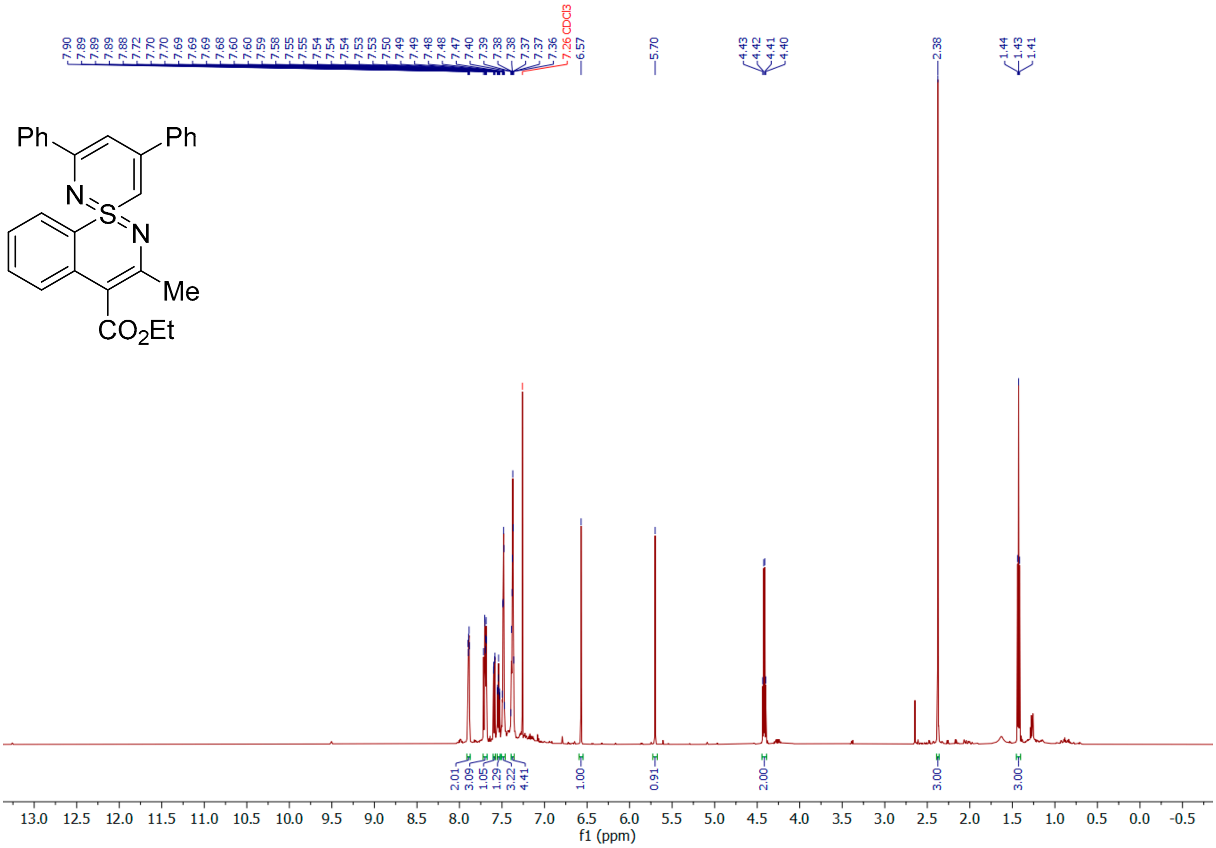

Figure S2: **^{1}H NMR** (600 MHz, $CDCl_3$) spectrum of **1**.

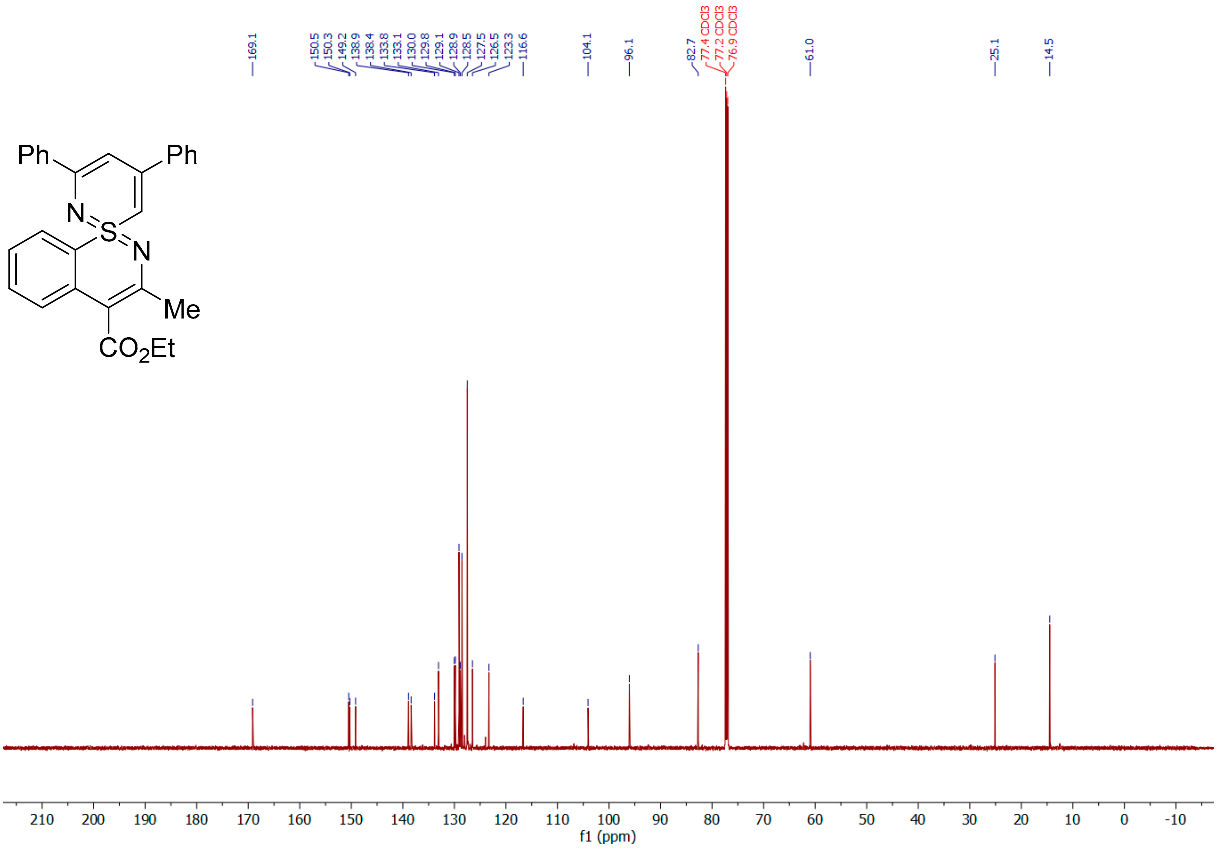

Figure S3: **^{13}C {^{1}H} NMR** (151 MHz, $CDCl_3$) spectrum of **1**.

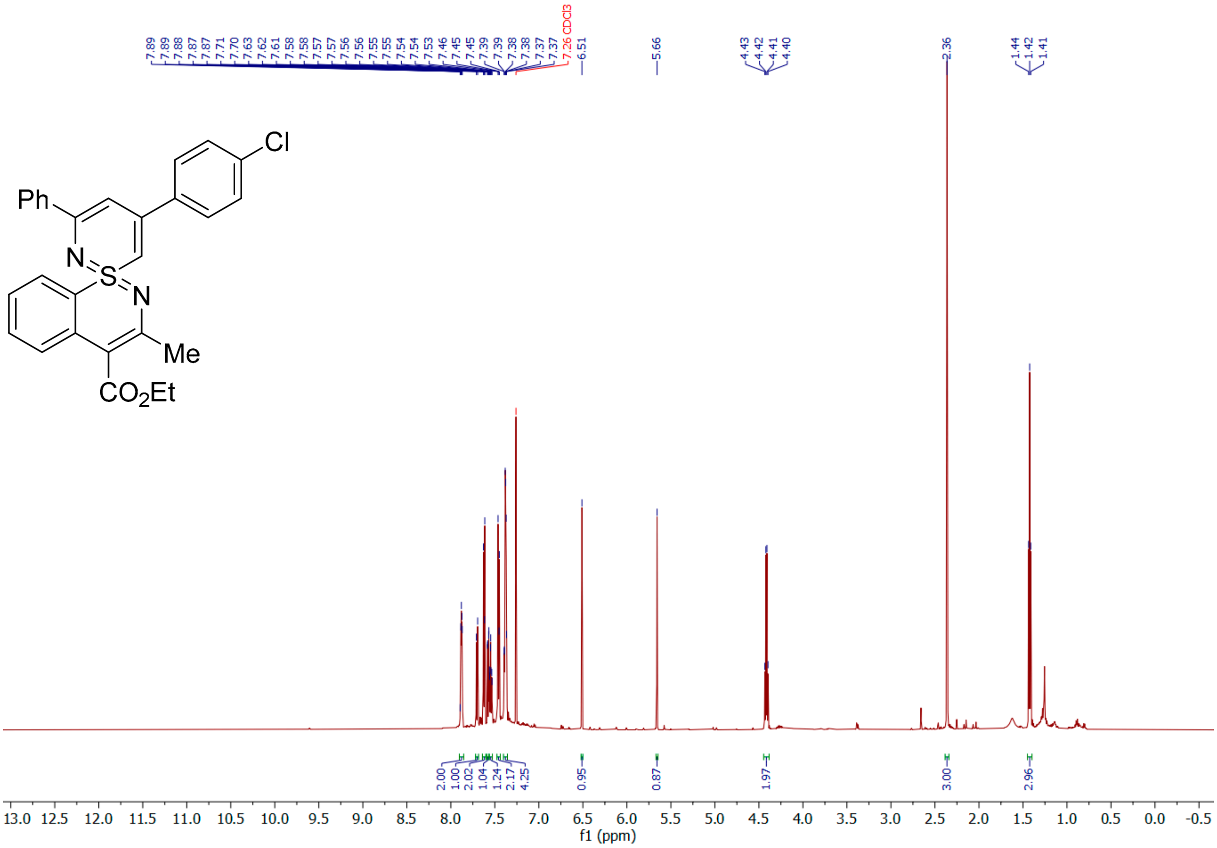

Figure S4: **¹H NMR** (600 MHz, $CDCl_3$) spectrum of **2**.

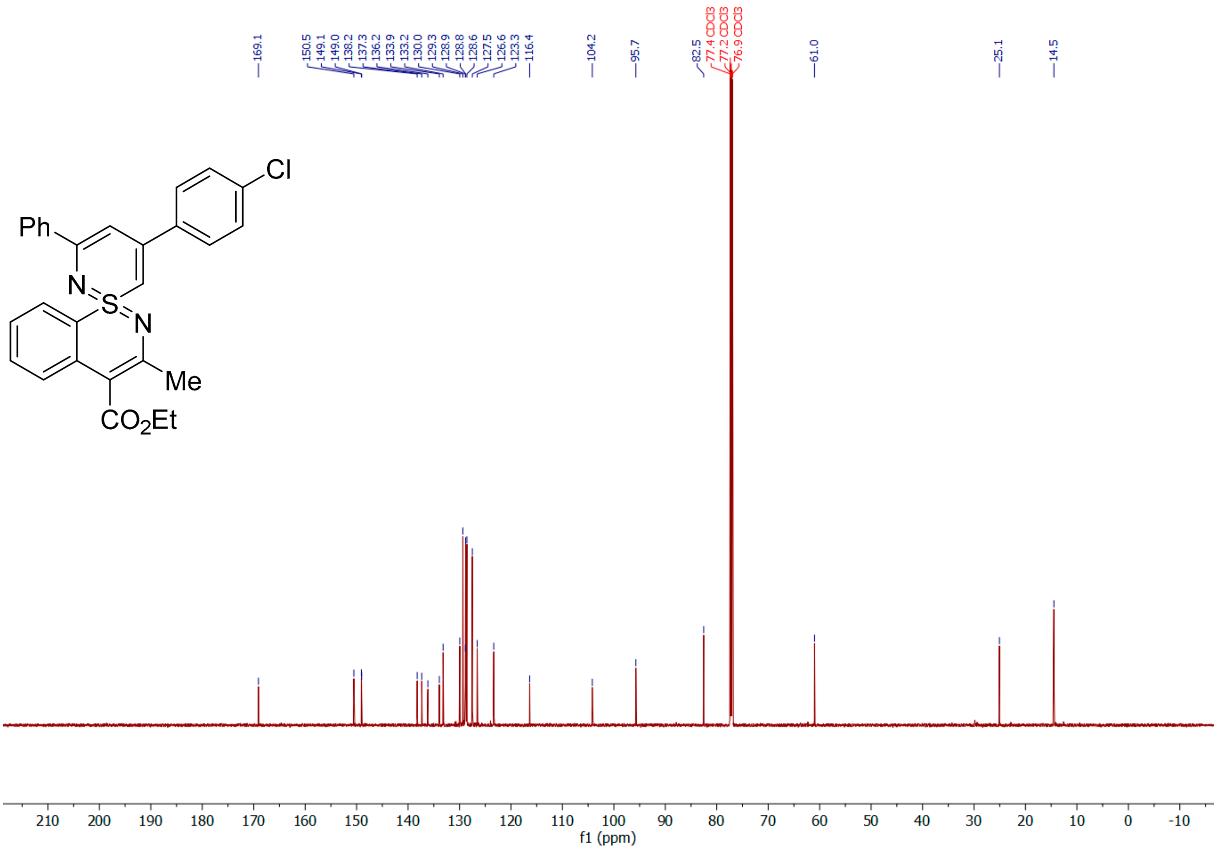

Figure S5: **^{13}C {^{1}H} NMR** (151 MHz, $CDCl_3$) spectrum of **2**.

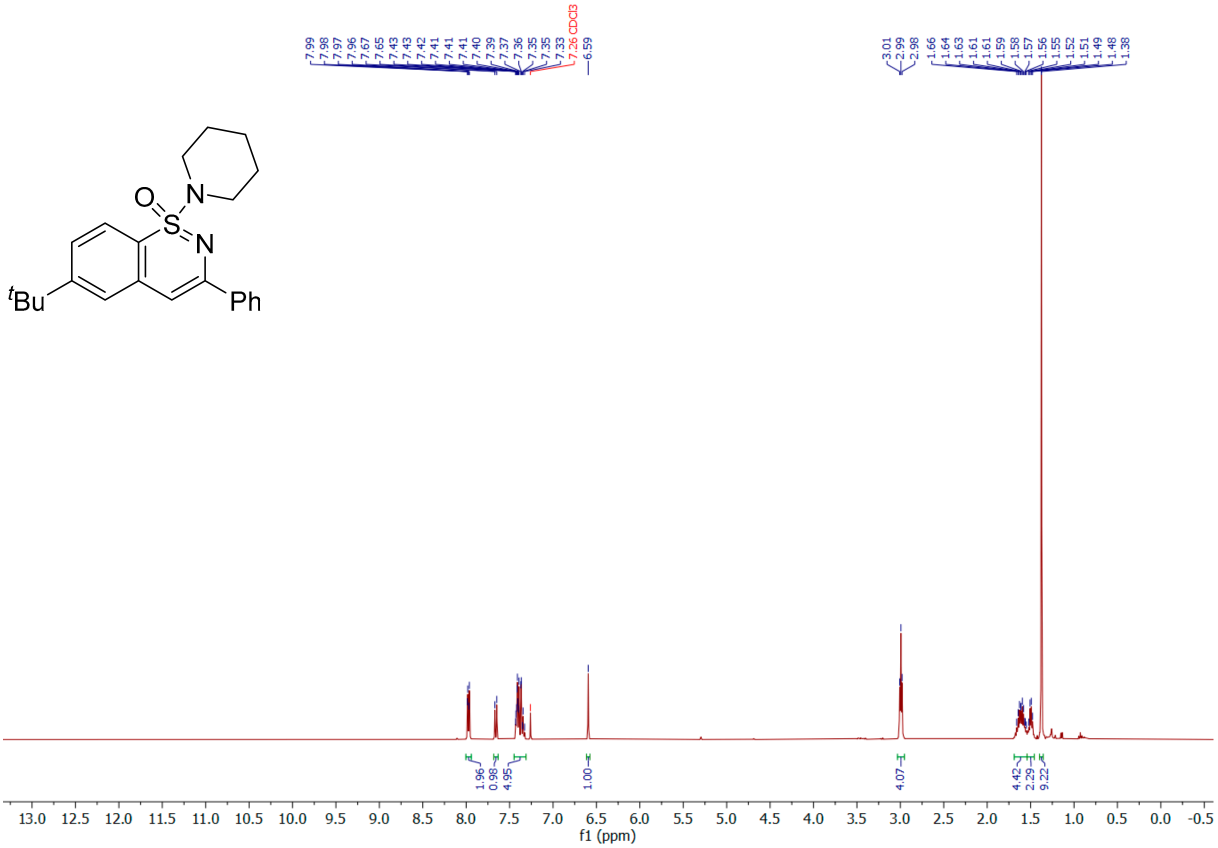

Figure S6: ^{1}H NMR (400 MHz, $CDCl_3$) spectrum of **9**.

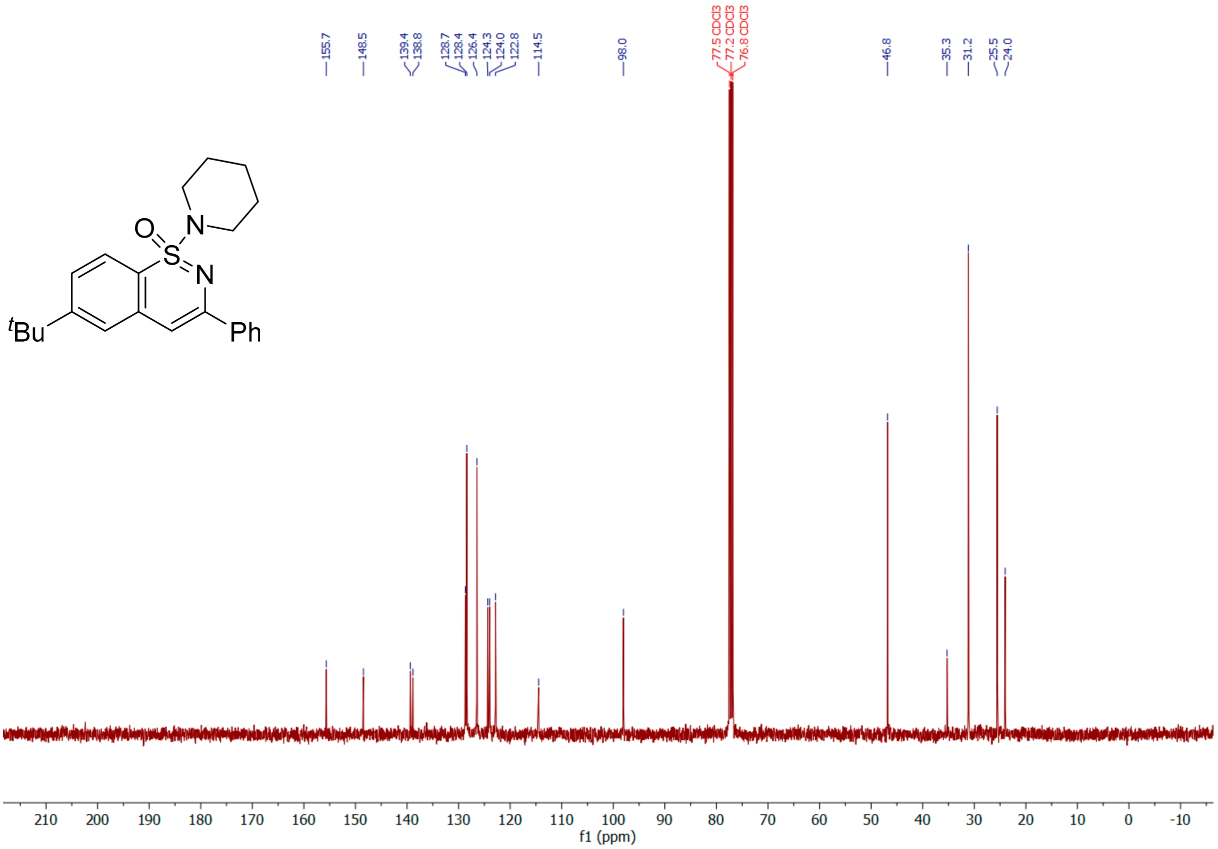

Figure S7: ^{13}C {^{1}H} NMR (101 MHz, $CDCl_3$) spectrum of **9**.

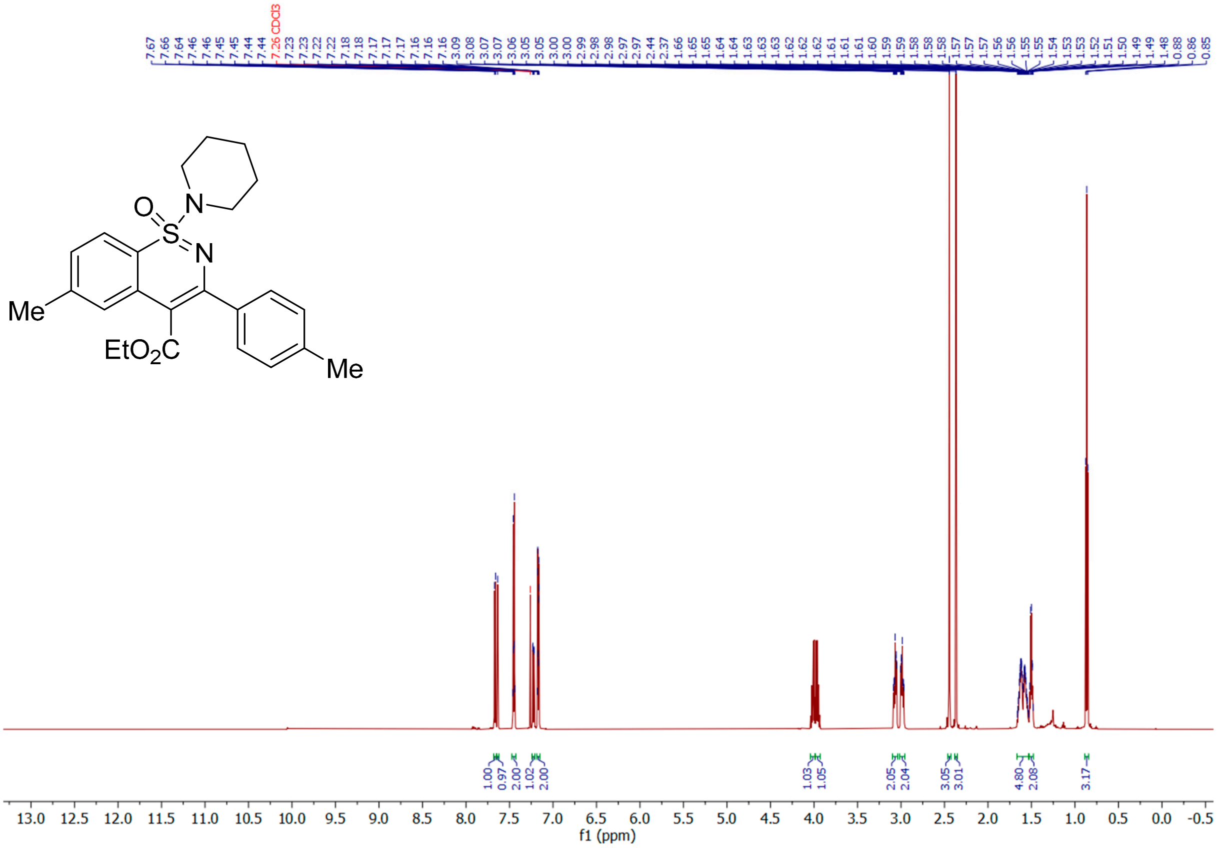

Figure S8: ^{1}H NMR (600 MHz, $CDCl_3$) spectrum of **17**.

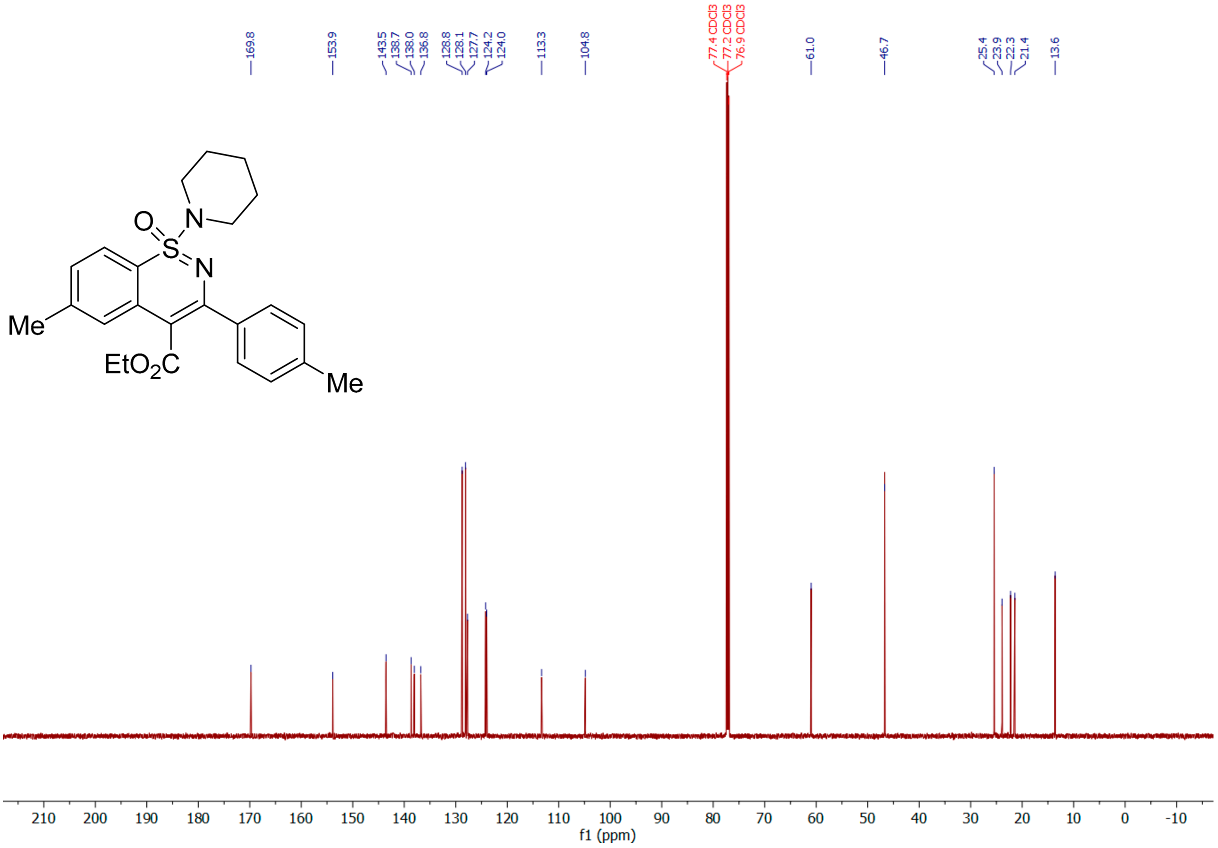

Figure S9: ^{13}C {^{1}H} NMR (151 MHz, $CDCl_3$) spectrum of **17**.

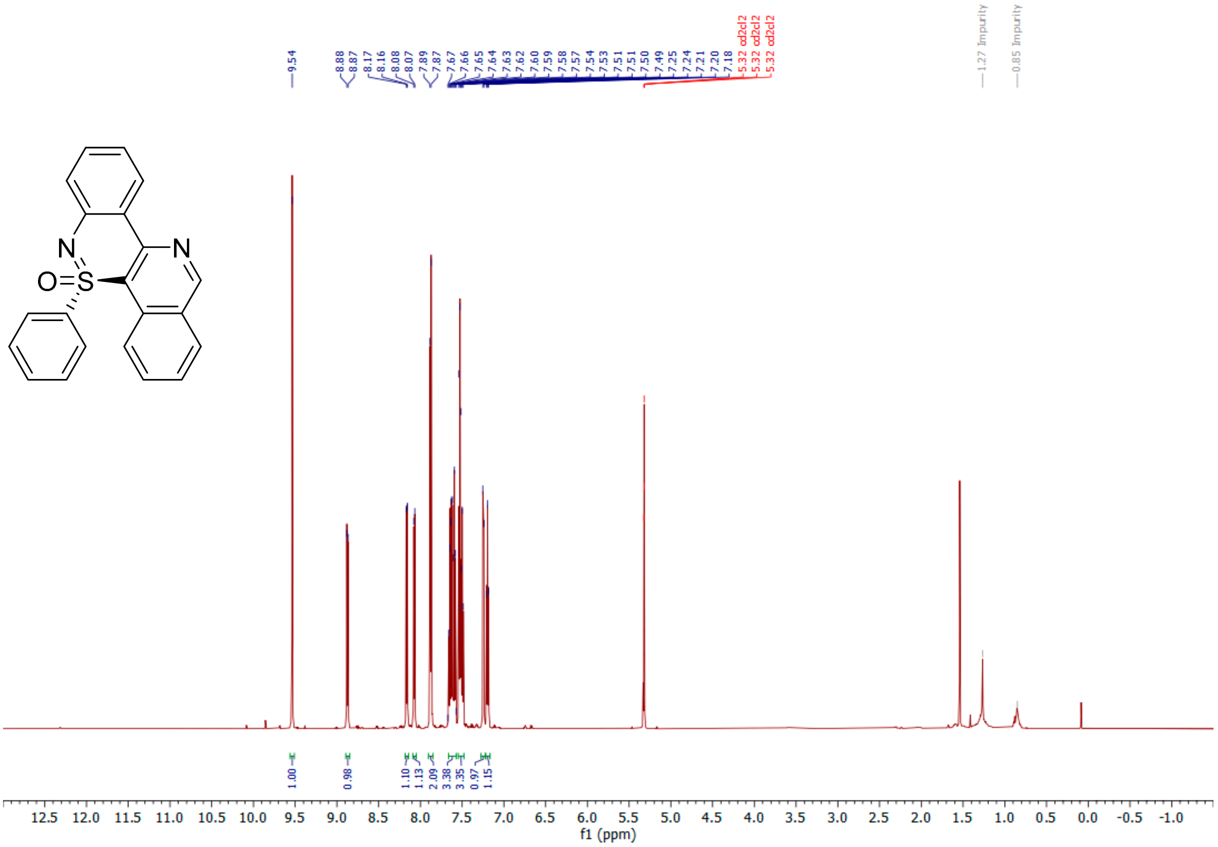

Figure S10: ^{1}**H NMR** (600 MHz, CD_2Cl_2) spectrum of **23**.

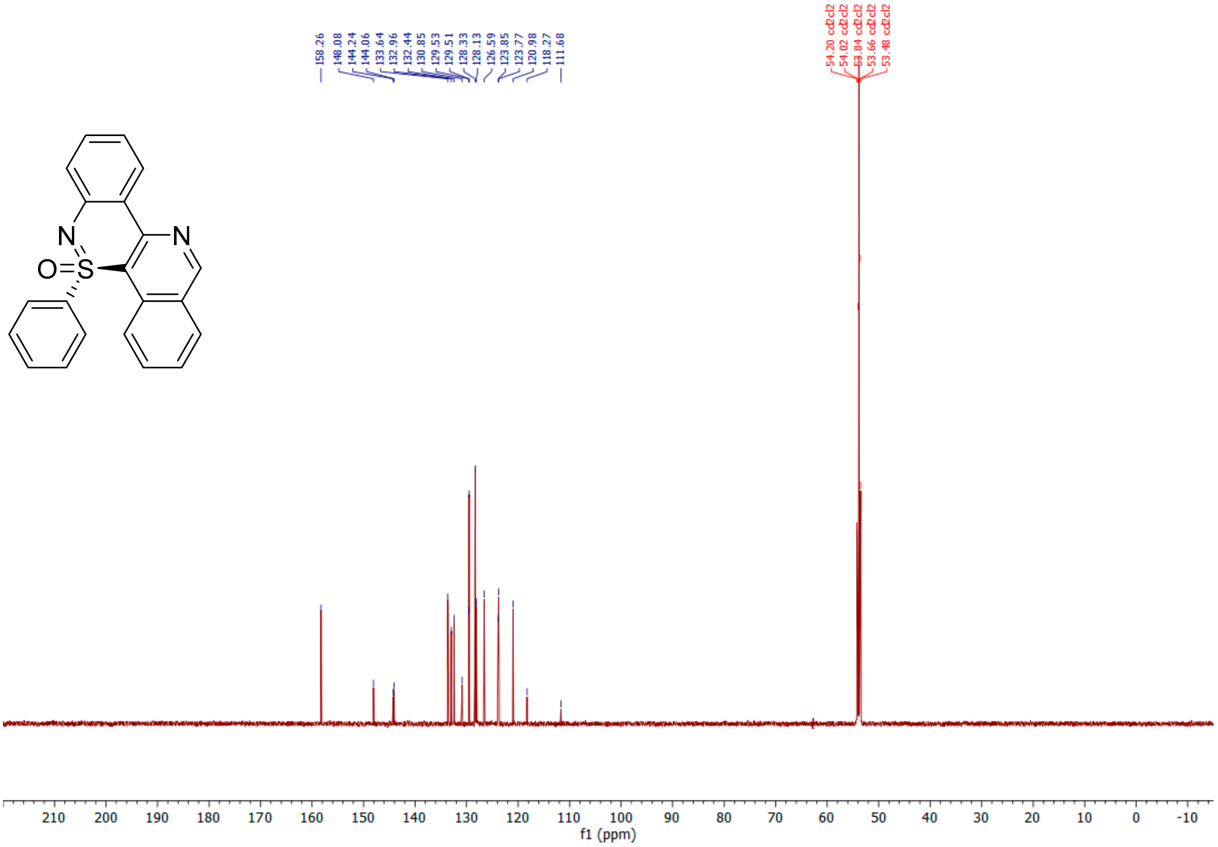

Figure S11: ^{13}**C {^{1}H} NMR** (151 MHz, CD_2Cl_2) spectrum of **23**.

5. CSP-HPLC Chromatograms

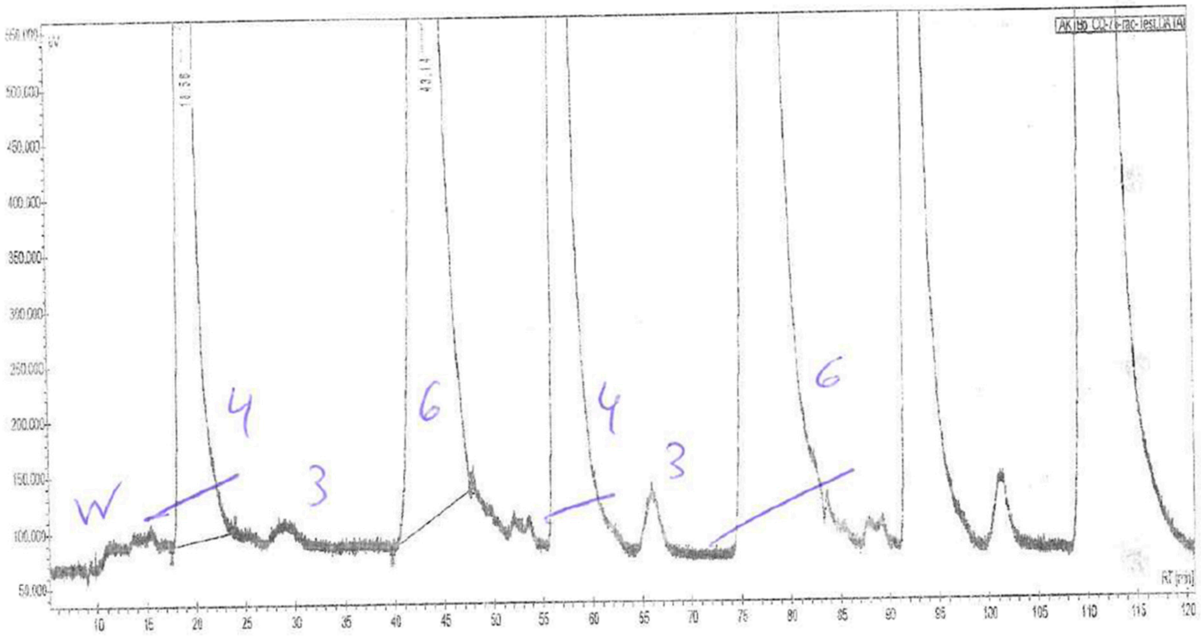

AK Bo CQ-76-rac-Test.DATA [UV Kanal]

Index	Name	Time [Min]	Quantity [% Area]	Height [µV]	Area [µV Min]	Area % [%]
1	UNKNOWN	18,56	39,58	913667,4	1929783,2	39,581
2	UNKNOWN	43,14	60,42	897390,7	2945802,6	60,419
Total			100,00	1811058,1	4875585,8	100,000

Figure S12: Preparative CSP-HPLC chromatogram for the separation of ***rac*-9**.

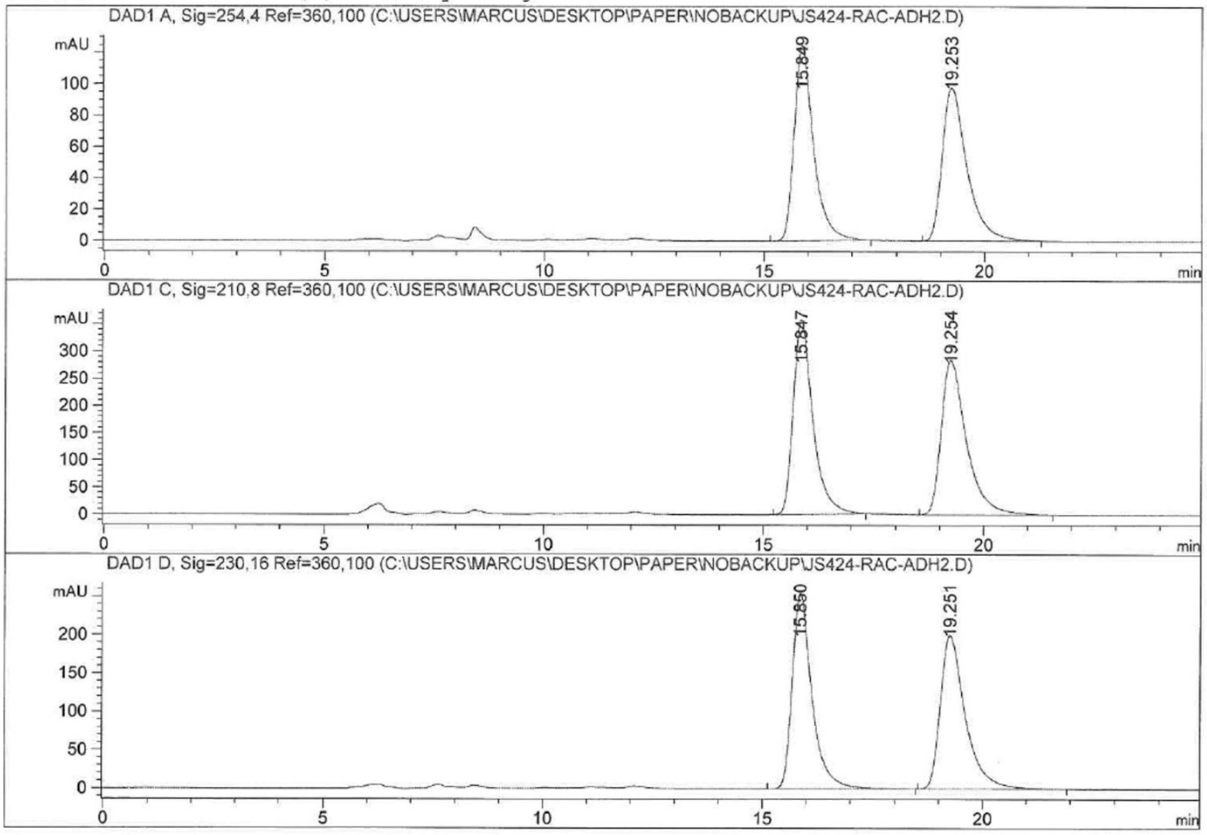

Signal 1: DAD1 A, Sig=254,4 Ref=360,100

Peak #	RetTime [min]	Type	Width [min]	Area [mAU*s]	Height [mAU]	Area %
1	15.849	BB	0.4620	3846.39160	124.66165	49.9929
2	19.253	BB	0.5694	3847.48071	97.71768	50.0071
Totals :				7693.87231	222.37933	

Signal 2: DAD1 C, Sig=210,8 Ref=360,100

Peak #	RetTime [min]	Type	Width [min]	Area [mAU*s]	Height [mAU]	Area %
1	15.847	BB	0.4688	1.11943e4	358.02304	49.6466
2	19.254	BB	0.5542	1.13536e4	284.11115	50.3534
Totals :				2.25479e4	642.13419	

Signal 3: DAD1 D, Sig=230,16 Ref=360,100

Peak #	RetTime [min]	Type	Width [min]	Area [mAU*s]	Height [mAU]	Area %
1	15.850	BB	0.4647	7974.35986	255.06531	50.1747
2	19.251	BB	0.5939	7918.84033	200.00279	49.8253
Totals :				1.58932e4	455.06810	

Figure S13: Analytical CSP-HPLC chromatogram of ***rac*-9**.

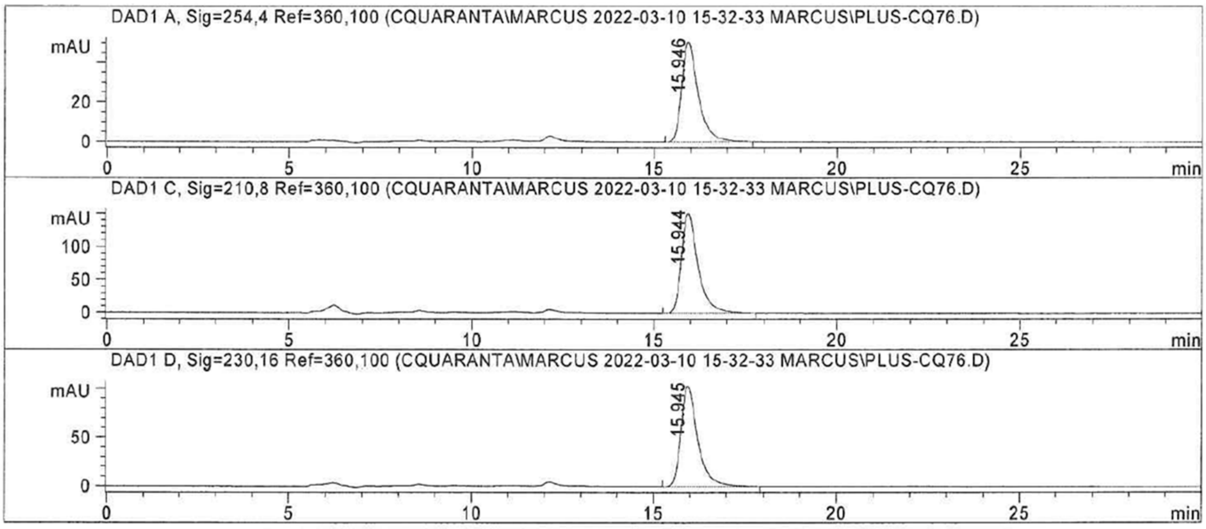

DAD1 A, Sig=254,4 Ref=360,100

Peak #	Ret. Time in min	Width in min	Height in mAU	Area in mAU*s	Area %
1	15.946	0.5234	50.55218	1587.41309	100.0000
Total				1587.41309	100.0000

DAD1 C, Sig=210,8 Ref=360,100

Peak #	Ret. Time in min	Width in min	Height in mAU	Area in mAU*s	Area %
1	15.944	0.5279	150.81110	4776.72998	100.0000
Total				4776.72998	100.0000

DAD1 D, Sig=230,16 Ref=360,100

Peak #	Ret. Time in min	Width in min	Height in mAU	Area in mAU*s	Area %
1	15.945	0.5272	103.02607	3258.79297	100.0000
Total				3258.79297	100.0000

Figure S14: Analytical CSP-HPLC chromatogram of **(+)-9**.

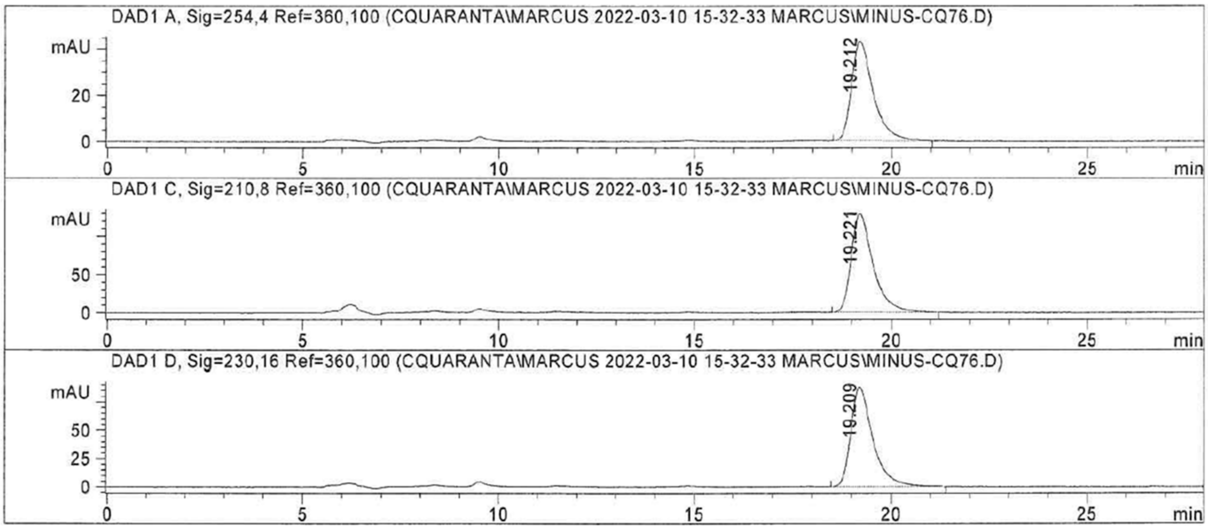

DAD1 A, Sig=254,4 Ref=360,100

Peak #	Ret. Time in min	Width in min	Height in mAU	Area in mAU*s	Area %
1	19.212	0.6426	42.86566	1652.70715	100.0000
Total				1652.70715	100.0000

DAD1 C, Sig=210,8 Ref=360,100

Peak #	Ret. Time in min	Width in min	Height in mAU	Area in mAU*s	Area %
1	19.221	0.6507	128.72009	5025.45654	100.0000
Total				5025.45654	100.0000

DAD1 D, Sig=230,16 Ref=360,100

Peak #	Ret. Time in min	Width in min	Height in mAU	Area in mAU*s	Area %
1	19.209	0.6485	87.89169	3419.79272	100.0000
Total				3419.79272	100.0000

Figure S15: Analytical CSP-HPLC chromatogram of **(–)-9**.

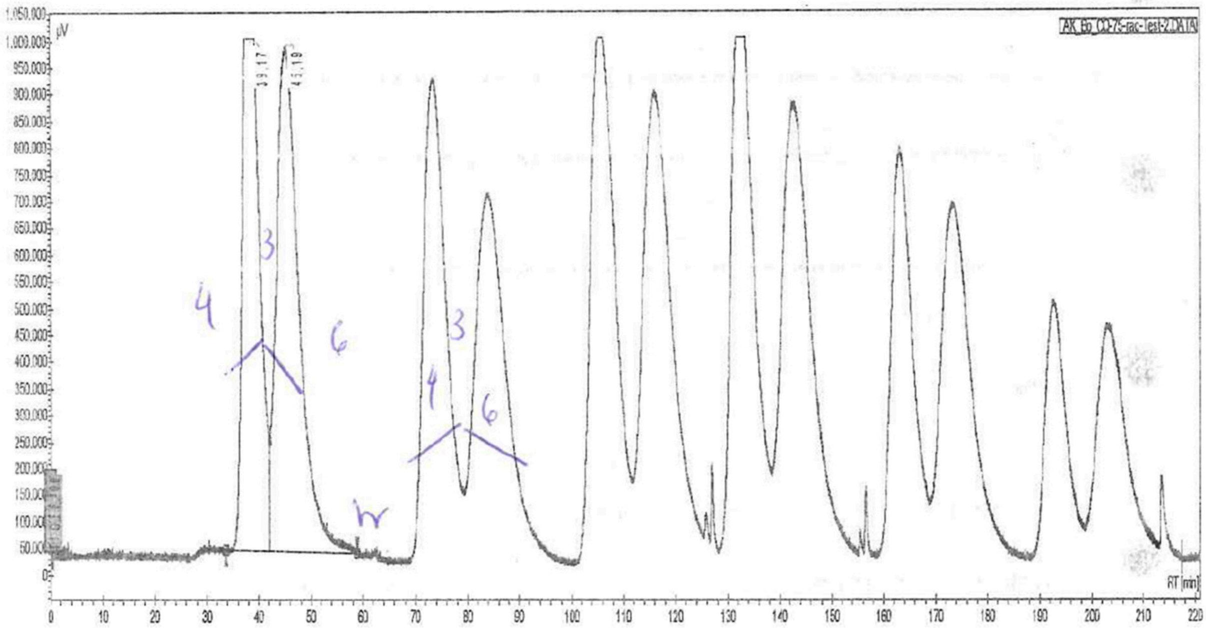

AK_Bo_CQ-75-rac-Test-2.DATA [UV Kanal]

Index	Name	Time [Min]	Quantity [% Area]	Height [µV]	Area [µV.Min]	Area % [%]
1	UNKNOWN	39.17	45.03	957403.6	3897952.1	45.032
2	UNKNOWN	45.19	54.97	941483.1	4757930.2	54.968
Total			100.00	1898886.7	8655882.3	100.000

Figure S16: Preparative CSP-HPLC chromatogram for the separation of ***rac*-17**.

Additional Info : Peak(s) manually integrated

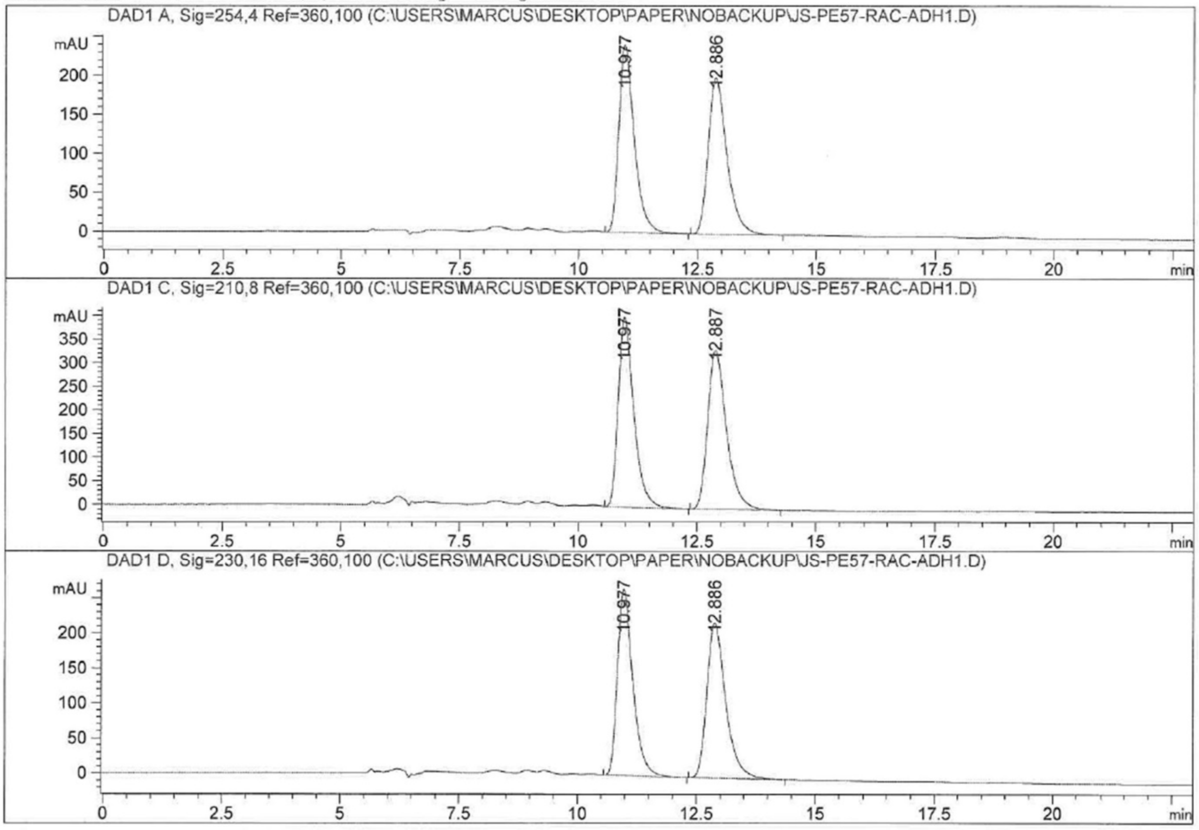

Signal 1: DAD1 A, Sig=254,4 Ref=360,100

Peak #	RetTime [min]	Type	Width [min]	Area [mAU*s]	Height [mAU]	Area %
1	10.977	VB	0.3375	5442.67139	240.98395	50.0226
2	12.886	BB	0.4046	5437.75049	200.40228	49.9774

Totals : 1.08804e4 441.38623

Signal 2: DAD1 C, Sig=210,8 Ref=360,100

Peak #	RetTime [min]	Type	Width [min]	Area [mAU*s]	Height [mAU]	Area %
1	10.977	VB	0.3418	9170.69238	402.50711	50.0131
2	12.887	BB	0.4083	9165.89941	335.97614	49.9869

Totals : 1.83366e4 738.48325

Signal 3: DAD1 D, Sig=230,16 Ref=360,100

Peak #	RetTime [min]	Type	Width [min]	Area [mAU*s]	Height [mAU]	Area %
1	10.977	BB	0.3387	5986.68457	265.84302	49.8100
2	12.886	BB	0.4096	6032.35205	221.60083	50.1900

Totals : 1.20190e4 487.44385

Figure S17: Analytical CSP-HPLC chromatogram of ***rac*-17**.

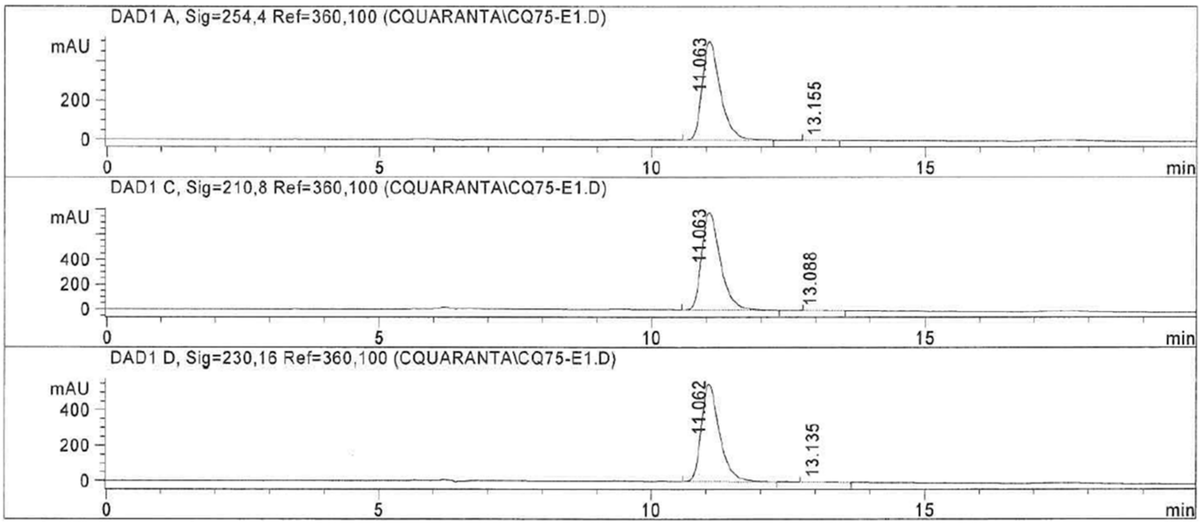

DAD1 A, Sig=254,4 Ref=360,100

Peak #	Ret. Time in min	Width in min	Height in mAU	Area in mAU*s	Area %
1	11.063	0.3875	500.98926	11648.96777	99.8250
2	13.155	0.4079	0.83453	20.42238	0.1750
Total				11669.39015	100.0000

DAD1 C, Sig=210,8 Ref=360,100

Peak #	Ret. Time in min	Width in min	Height in mAU	Area in mAU*s	Area %
1	11.063	0.4016	783.58191	18880.19336	99.8017
2	13.088	0.3917	1.59587	37.50695	0.1983
Total				18917.70031	100.0000

DAD1 D, Sig=230,16 Ref=360,100

Peak #	Ret. Time in min	Width in min	Height in mAU	Area in mAU*s	Area %
1	11.062	0.3878	551.65228	12836.75879	99.7995
2	13.135	0.4159	1.03333	25.78634	0.2005
Total				12862.54513	100.0000

Figure S18: Analytical CSP-HPLC chromatogram of **(+)-17**.

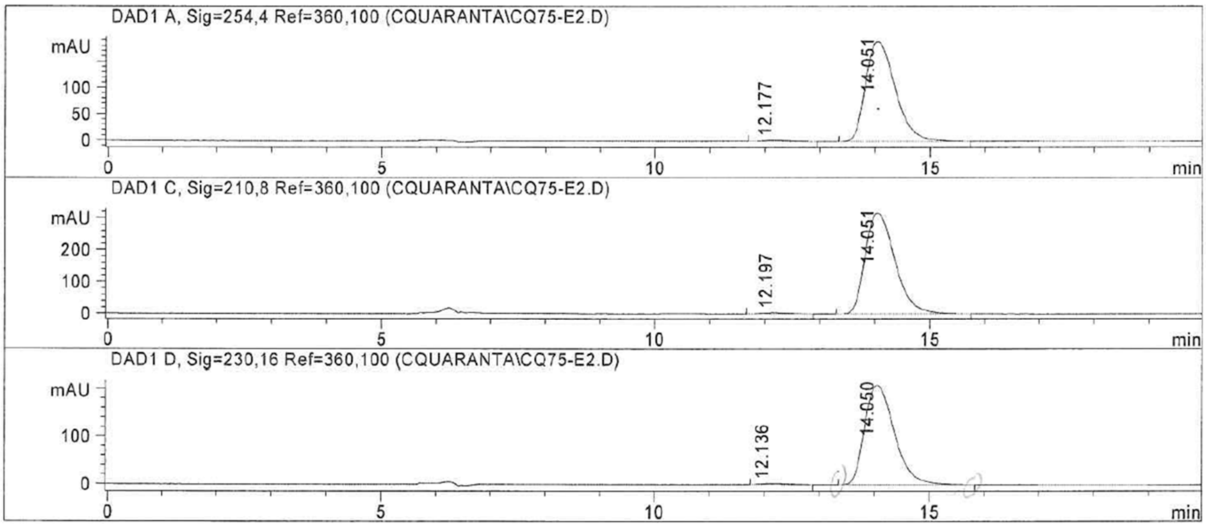

DAD1 A, Sig=254,4 Ref=360,100

Peak #	Ret. Time in min	Width in min	Height in mAU	Area in mAU*s	Area %
1	12.177	0.4017	1.87549	63.60449	0.8930
2	14.051	0.6202	189.67519	7058.66943	99.1070
Total				7122.27392	100.0000

DAD1 C, Sig=210,8 Ref=360,100

Peak #	Ret. Time in min	Width in min	Height in mAU	Area in mAU*s	Area %
1	12.197	0.3953	3.11188	103.31315	0.8655
2	14.051	0.6217	317.24164	11833.05957	99.1345
Total				11936.37272	100.0000

DAD1 D, Sig=230,16 Ref=360,100

Peak #	Ret. Time in min	Width in min	Height in mAU	Area in mAU*s	Area %
1	12.136	0.4028	1.91291	63.41581	0.8106
2	14.050	0.6199	208.62512	7759.91797	99.1894
Total				7823.33377	100.0000

Figure S19: Analytical CSP-HPLC chromatogram of **(–)-17**.
